# A Critical Review on the Standardization and Quality Assessment of Nonfunctional Laboratory Tests Frequently Used to Identify Inborn Errors of Immunity

**DOI:** 10.3389/fimmu.2021.721289

**Published:** 2021-11-09

**Authors:** Sandro Félix Perazzio, Patricia Palmeira, Dewton Moraes-Vasconcelos, Andréia Rangel-Santos, João Bosco de Oliveira, Luis Eduardo Coelho Andrade, Magda Carneiro-Sampaio

**Affiliations:** ^1^ Division of Rheumatology, Universidade Federal de São Paulo, Sao Paulo, Brazil; ^2^ Immunology Division, Fleury Medicine and Health Laboratory, Sao Paulo, Brazil; ^3^ Laboratório de Investigação Médica (LIM-36), Hospital das Clinicas da Faculdade de Medicina da Universidade de São Paulo (FMUSP), Sao Paulo, Brazil; ^4^ Laboratório de Investigação Médica (LIM-56), Hospital das Clinicas da Faculdade de Medicina da Universidade de São Paulo (FMUSP), Sao Paulo, Brazil; ^5^ Division of Genetics, Hospital Israelita Albert Einstein, Sao Paulo, Brazil; ^6^ Department of Pediatrics, Faculdade de Medicina da Universidade de São Paulo (FMUSP), Sao Paulo, Brazil

**Keywords:** inborn errors of immunity, primary immunodeficiencies, quality assessment (QAS), standardization, reference range

## Abstract

Inborn errors of immunity (IEI), which were previously termed primary immunodeficiency diseases, represent a large and growing heterogeneous group of diseases that are mostly monogenic. In addition to increased susceptibility to infections, other clinical phenotypes have recently been associated with IEI, such as autoimmune disorders, severe allergies, autoinflammatory disorders, benign lymphoproliferative diseases, and malignant manifestations. The IUIS 2019 classification comprises 430 distinct defects that, although rare individually, represent a group affecting a significant number of patients, with an overall prevalence of 1:1,200-2,000 in the general population. Early IEI diagnosis is critical for appropriate therapy and genetic counseling, however, this process is deeply dependent on accurate laboratory tests. Despite the striking importance of laboratory data for clinical immunologists, several IEI-relevant immunoassays still lack standardization, including standardized protocols, reference materials, and external quality assessment programs. Moreover, well-established reference values mostly remain to be determined, especially for early ages, when the most severe conditions manifest and diagnosis is critical for patient survival. In this article, we intend to approach the issue of standardization and quality control of the nonfunctional diagnostic tests used for IEI, focusing on those frequently utilized in clinical practice. Herein, we will focus on discussing the issues of nonfunctional immunoassays (flow cytometry, enzyme-linked immunosorbent assays, and turbidimetry/nephelometry, among others), as defined by the pure quantification of proteins or cell subsets without cell activation or cell culture-based methods.

## Introduction

Primary immunodeficiency diseases (PID) constitute a large and fast-growing heterogeneous group of genetic diseases, mostly (but not exclusively) caused by loss or gain of function germline mutations. Although PID are classically manifested as increased susceptibility to infections, recently, an increasing variety of autoimmune, autoinflammatory, allergic, and malignant phenotypes has also been recognized (1). This comprehensive concept was closely accompanied by a broader pathophysiological understanding of such disorders, which are now grouped in the category of inborn errors of immunity (IEI) (2). Despite individual rarity, IEI collectively represent a significant proportion of patients, with an estimated overall prevalence of 1:1,200-2,000 (3, 4). They now comprise 406 distinct disorders with 430 different gene defects subdivided into categories listed in the 2019 International Union of Immunological Societies (IUIS) classification (5, 6), approximately two-thirds of which were recognized in the past decade (**Table 1**). As evidence of dynamic development in the area, 26 additional monogenic gene defects have recently been reported and may soon be included in the IEI portfolio (8).

**Table 1:** IEI categories and clinical prototypes according to the 2019 IUIS update of the phenotypical classification (6).

Category	Clinical phenotypes (n)	Clinical prototypes	Causative genes (n)	% of total IEI (7)
Immunodeficiencies affecting cellular and humoral immunity	58	SCID	59	7
Combined immunodeficiencies with associated or syndromic features	68	Wiskott Aldrich syndrome, DiGeorge syndrome, Bloom syndrome, ataxia telangiectasia, hyper-IgE syndrome	63	11
Predominantly antibody deficiencies	48	Agammaglobulinemia, CVID	40	57
Diseases of immune dysregulation	46	HLH, ALPS, IPEX, APECED	45	6
Congenital defects of phagocyte number or function	41	CGD, LAD	41	8
Defects in intrinsic and innate immunity	64	CMC, MSMD, recurrent HSE	67	2
Autoinflammatory disorders	43	FMF, CAPS, TRAPS, MVKD, PAPA syndrome, type 1 interferonopathies	42	3
Complement deficiencies	27	Complement components deficiencies, hereditary angioedema	33	2
Bone marrow failure	8	Fanconi anemia, dyskeratosis congenita	40	3

The total numbers of clinical phenotypes and causative genes are also represented for each category. Of note, these two variables are not always identical due to the presence of different clinical phenotypes caused by a single gene and vice versa. The frequencies of each representative category within the total number of IEI patients according to the Latin American Society of Immunodeficiencies (7) are also depicted.

ALPS, autoimmune lymphoproliferative syndrome; APECED, autoimmune polyendocrinopathy with candidiasis and ectodermal dystrophy; CAPS, cryopyrin-associated periodic syndrome; CGD, chronic granulomatous disease; CMC, chronic mucocutaneous candidiasis; CVID, common variable immunodeficiency; FMF, familial Mediterranean fever; HLH, hemophagocytic lymphohistiocytosis; HSE, Herpes simplex encephalitis; IEI, inborn errors of immunity; IPEX, immune dysregulation, polyendocrinopathy, enteropathy X-linked; IUIS, International Union of Immunology Societies; LAD, leukocyte adhesion deficiency; MSMD, Mendelian susceptibility to mycobacterial disease; MVKD, mevalonate kinase deficiency; PAPA, pyogenic sterile arthritis, pyoderma gangrenosum, acne; SCID, severe combined immunodeficiency; TRAPS, TNF receptor-associated periodic syndrome.

Early specific diagnosis of IEI patients is deeply dependent on accurate lab tests, and is pivotal for targeted therapy and appropriate patient and familial genetic counseling (2). In this context, the laboratory serves as the primary source of diagnostic information used to define the underlying immunologic defect (9). Clinically guided IEI laboratory investigations may follow three main consecutive steps: screening, advanced testing, and molecular confirmation (**Table 2**). Flow cytometry assays and molecular analyses are currently the most relevant methodological platforms in the area. Moreover, functional tests are critical for identifying particular IEI. Some assays are simple and disseminated worldwide, while others are only available in specific research centers, creating an obstacle for knowledge spread in the area.

**Table 2:** Suggested IEI clinically guided laboratory investigation guidelines, according to three proposed main consecutive steps: screening, advanced tests, and molecular confirmation.

**Screening**	
Cell blood count and peripheral smear Serum immunoglobulins (IgG, IgA, IgM and total IgE) Vaccine response against polysaccharide (e.g.: *Streptococcus pneumoniae*) and protein antigens (e.g.: tetanus toxoid); spontaneous specific antibodies (anti-blood group Abs, isohemagglutinins) Peripheral blood basic immunophenotyping: CD3, CD4, CD8, CD19, and CD16/CD56 Complement system assessment: CH50 and AH50 Phagocyte oxidative burst: dihydrorhodamine oxidation assay TREC copies	
**Advanced tests**	
Predominantly antibody deficiencies	IgG subclasses Vaccine response against neoantigens (bacteriophage, *Salmonella typhi* capsular polysaccharide vaccine) B cell immunophenotyping Intracellular protein expression – BTK
Combined immunodeficiencies affecting cellular and humoral immunity	Chromosomal instability T cell immunophenotyping (flow cytometry) CD40/CD40L binding assay Cell surface protein expression – CD132 (IL-2Rγ), CD127 (IL-7Rα), MHC I and II Intracellular protein expression – WASP (Wiskott-Aldrich protein) Lymphoproliferation in response to mitogens, alloantigens and recall antigens TCR repertoire analysis: TCR-CDR3 spectratyping and flow cytometry-based TCR Vβ frequency Adenosine deaminase and PNP activity *In vitro* cytokine production in cell culture supernatant
Diseases of immune dysregulation	NK cytotoxic activity assay and CD107a degranulation Soluble CD25 Intracellular protein expression (PRF1, SAP/SH2DIA, XIAP) Double negative TCRα/β circulating T cells Lymphocyte apoptosis assay Soluble mediators: IL-10, IL-18, soluble FASL and vitamin B12 T regulatory cells (CD4+/CD25+/CD127low/Foxp3+) number and function STAT1 phosphorylation assay CTLA-4 functional testing
Defects in intrinsic and innate immunity	IL-12/IFNγ axis functional assay Intracellular protein expression: IFNγ-R1 and IFNγ-R2 Cell surface protein expression: CD18, CD11a/CD11b/CD11c, CD15 IκBα degradation TLR functional assays (CD62L shedding)
Autoinflammatory disorders	Type 1 interferon signature Serum IgD Urinary mevalonic acid
Complement deficiencies	Specific complement components
**Genetic and molecular tests**	
Karyotype, FISH, MLPA, copy number variation analysis Specific single gene-sequencing (Sanger) Next-generation sequencing (panels, whole-exome or genome sequencing)	

A list of the main nonfunctional IEI diagnostic tests is provided and should be individually considered according to the stage of investigation.

AH50, total hemolytic complement (alternative pathway); ALPS, autoimmune lymphoproliferative syndrome; CH50, total hemolytic complement (classic pathway); FISH, fluorescence in situ hybridization; IEI, inborn errors of immunity; MLPA, multiplex ligation-dependent probe amplification; TCR, T cell receptor; TLR, Toll-like receptors; TREC, T cell receptor excision circle.

Despite the striking importance of laboratory data for clinical immunologists, several IEI relevant immunoassays still lack standardization, including standardized protocols, reference materials, and external quality assessment programs. Moreover, well-established reference values mostly remain to be determined, especially for early ages, when the most severe conditions manifest and diagnosis is critical for patient survival (10). Compared to biochemical tests, standardization and quality controls in immunoassays are rudimentary, partially due to the particular complexity of analytes. Immunoassays usually assess heterogeneous molecules, such as serum polyclonal antibodies, that share common characteristics, but are in fact distinct analytes with individual features.

The above-described issues reinforce the necessity of a parallel healthy control blood sample in some IEI diagnostic-driven nonfunctional immunoassays, assuming a high number of uncontrolled variables. This is particularly problematic in young patients whose blood is usually compared with adult control samples. Although challenging, tests for the identification of IEI need better standardization to improve the diagnostic accuracy. Such a hard task has precedents in other areas, such as the prothrombin activity assay, which, in the near past, was totally uncontrolled and is currently standardized within an international normalized ratio.

In this article, we will approach the issues of methodological standardization (including the definition of reference ranges) and quality control programs for nonfunctional tests used to identify IEI, focusing on those frequently utilized in clinical practice. We expect to not only contribute to critical lab result interpretation in bedside clinical evaluations, but also encourage clinical pathologists and researchers to improve the accuracy, reproducibility, and international harmonization of tests relevant to IEI diagnoses. Herein, we will focus on listing all papers addressing standardization and quality assessment programs and discussing the issues of nonfunctional immunoassays (flow cytometry, enzyme-linked immunosorbent assay, and turbidimetry/nephelometry, among others), as defined by the pure quantification of immunological critical molecules or cell subsets without the involvement of cell activation assays or cell culture-based methods.

Single-analyte quantification and flow cytometry-based assessments of the cell surface and intracellular protein expression will be considered nonfunctional tests in our paper. This category consists of both screening (e.g., immunoglobulin serum levels, specific serologies, T cell receptor excision circle quantification, etc.) and advanced tests (e.g., immunophenotyping panels, specific surface, and intracellular protein expression, among others). Cell activation and cell culture-based assays are considered “functional tests” and will not be approached here.

## Study Method

A broad search of the Medline/Pubmed, Google Scholar and Scielo databases was performed using the terms “reference range”, “standardization”, “quality assessment”, “quality control” and “QAS” crossed with all captions representing each IEI subarea below: “predominantly antibody deficiencies”, “IgG/IgM/IgA serum levels”, and “B/T cell immunophenotyping”, among others. The nonsystematic review included every paper approaching any methodological standardization and quality control programs.

## Regulatory Agencies and Lab Certification

Current regulation policies demand analytical validity reviews of great depth and scope for any newly developed test system prior to marketing, and, therefore, prior to use with patient specimens in the clinical diagnosis or treatment context. This process is usually performed and regulated by different national agencies (e.g., Food and Drug Administration, European Medicines Agency, Brazilian Health Regulatory Agency, etc.) hence, its validity is specific to the home country, although some nations eventually adopt foreign reviews. Safety and effectiveness assessments of the novel test system may also include the accuracy with which the test identifies, measures, or predicts the presence or absence of a clinical condition in a patient, constituting a process usually called clinical validity testing. In summary, regulatory agencies ensure that new devices intended for the diagnosis, treatment, or prevention of disease are safe and effective.

On the other hand, quality assessment programs are designed to regulate laboratories that perform testing on patient specimens to ensure accurate and reliable test results. Programs are usually based on regular routine surveys that certify participant labs with governmental or non-governmental institution approval [e.g., Clinical Laboratory Improvement Amendments (CLIA), College of American Pathologists (CAP), Brazilian Clinical Laboratory Accreditation Program, etc.]. Ultimately, the institutions assess the performance characteristics of a test to describe the quality of patient test results, including analyses of accuracy, precision, analytical sensitivity, analytical specificity, reportable range, reference interval, and any other performance characteristics required by the test system in the laboratory that intends to use it. In addition, regulatory requirements vary according to the equipment used and type of test performed: the more complex the test is to perform, the more stringent the requirements. Therefore this analytical validation is limited to the specific conditions, staff, equipment and patient population of the particular laboratory, so the findings of these laboratory-specific analytical validations are not meaningful outside of the laboratory that performed the analysis.

Thus, the two regulatory schemes described above are different in focus, scope and purpose, but they are intended to be complementary. Of note, especially in the United States, when a laboratory develops a test system such as an in-house laboratory-developed test (LDT) without receiving FDA clearance or approval, CLIA rules prohibit the release of any test results prior to laboratory establishment of certain performance characteristics related to analytical validity for the use of that test system in the laboratory’s own environment. In summary, any novel diagnostic system or device requires strictly addressing the following parameters as they apply to regulatory agency approval: accuracy, trueness, precision, reproducibility, robustness, linearity, reportable range, reference range, interfering substances, analytic sensitivity/specificity, limit of detection/quantification, and clinical sensitivity/specificity (11).

## Predominantly Antibody Deficiency

Predominantly antibody deficiency (PAD) encompasses the most frequent IEI reported in numerous series worldwide (**Table 1**), representing 60-80% of IEI identified in adults (12). Screening tests include immunoglobulin serum levels (IgG, IgM and IgA), antibody responses to both protein and polysaccharide vaccine antigens, and total circulating mature B cell numbers (CD19^+^ or CD20^+^) (**Table 2**). B cell immunophenotyping and rarely ordered IgG subclass serum levels should be postponed until the second step (13, 14).

### IgG (and Subclasses), IgM, and IgA Serum Levels

Serum IgG, IgA and IgM levels are the most important screening tests for the initial assessment of humoral immunodeficiencies and are usually evaluated by nephelometry or turbidimetry, which provide good correlation indices and fast and highly reproducible results for quantification in serum and other fluids (e.g., cephalospinal fluid).

Most laboratories have reference values of these parameters for all age groups, which may vary according to different ethnic groups and across countries (15) and are accredited and highly controlled by CAP. Well-established immunoglobulin and IgG subclass levels within two standard deviations (SD) of the mean in age-matched controls are considered normal. In clinical practice, two distinct scenarios must always be investigated: i) IgG levels below 400 mg/dL in school children, adolescents, or adults; and ii) serum levels clearly below the age-adjusted reference range (95% CI) in infants or small children (16). Another aspect to be considered is that serum IgG levels in the initial months of life may be masked by maternal IgG transplacental transference. Therefore, a new assessment after six months of life, by which point maternal IgG has already been degraded, is mandatory (17).

It is largely established that in selective IgA deficiency (SIgAD), which is the most common pediatric antibody deficiency with incidence rates varying between 1:143 and 1:18,500 (18), the serum concentration is always less than 7 mg/dL associated with normal serum IgG and IgM levels. As IgA only reaches adult levels later in life, and SIgAD diagnosis can only be confirmed after four years of age (19).

IgG subclass ordering has restricted utility, and is thus not a consensus for IEI diagnosis, although it can be particularly useful in SIgAD associated with recurrent sinopulmonary infections (20–39).

### IgD

Serum IgD levels have usually been assessed by ELISA, with reports of a wide range among healthy individuals (0.10 to 213 μg/ml). Serum IgD concentrations have been shown to increase over childhood and decrease with age, but no normality range has been well-established for different age groups (40, 41).

IgD measurement is not usually included in a standard antibody evaluation; however, this analyte assessment is useful if there is a clinical suspicion of mevalonate kinase deficiency (MKD). In this monogenic autoinflammatory disease (*MVK*), serum polyclonal IgD concentrations are elevated, with a median of approximately 400 U/mL (1 U = 1.41 μg/mL). MKD is also called hyperimmunoglobulinemia D and periodic fever syndrome or hyper-IgD syndrome (HIDS), although the reason for the increased IgD concentrations and their role in pathogenesis have not yet been fully clarified (42, 43).

### Total IgE

Serum IgE levels are usually assessed by ELISA or fluorescent solid-phase immunoassay, however there are no well-established serum IgE reference values for different age groups, especially for healthy infants and children (44–46). IgE serum levels between 100 and 200 kU/L (1 U/L = 2,4 ng/ml) are considered normal for healthy adults (45). Longitudinal studies in “normal” children have demonstrated that IgE levels tend to progressively increase in the first decade of life, with large variability in early first years, followed by plateauing at age 10-13 years, and decreasing slightly in the following years (44).

Allergic disorders are the most frequent cause of high IgE levels, although parasitic infestations may also be relevant conditions in tropical areas (47–49). Regarding IEI, elevated serum IgE levels are associated with several diseases, such as: i) hyper-IgE syndrome (loss-of-function *STAT3* mutation); ii) Dedicator of CytoKinesis 8 (DOCK8) deficiency; iii) IPEX – Immunedysregulation Polyendocrinopathy Enteropathy X-linked syndrome (*FOXP3*); iv) Wiskott-Aldrich syndrome (*WAS*); v) Phosphoglucomutase 3 (*PGM3*) deficiency; vi) Comèl-Netherton syndrome (*SPINK5*); and vii) Loeys-Dietz syndrome (*TGFBR1*). Of interest, all of these conditions present severe allergic dermatitis (50–52). Elevated IgE levels also represent a characteristic finding in Omenn syndrome (*RAG1, RAG2, DCLRE1C* or *IL7R*), which is an extremely severe condition seen in some infants with severe combined immunodeficiency (SCID) (53, 54). Very high concentrations of IgE — above 1,000-2,000 kU/L at an early age — should direct attention to an IEI.

On the other hand, IgE deficiency (<2.5 kU/L), which has been considered without clinical consequences for decades, has recently been associated with higher rates and risks for the development of malignancies (55). IgE deficiency is also seen in some IEI, such as ataxia-telangiectasia (*ATM*), as well as in some patients with common variable immunodeficiency (CVID), SIgAD or IgG subclass deficiencies (56–58). Down syndrome patients usually present low total and specific IgE concentrations, even those presenting chronic or recurrent respiratory manifestations (59).

### Postimmunization Measurement of *In Vivo* Specific Antibody Responses

Specific antibody responses can be evaluated by testing for spontaneous specific antibodies, such as isohemagglutinins, as well as antibodies to documented previous immunizations or infections. The vaccine antibody response reflects an individual’s ability to respond specifically to antigens contained in the vaccine. Thus, we must separately consider vaccines containing polysaccharide antigens, protein antigens and polysaccharide-conjugated-to-protein antigens, asg only the B lymphocyte response is involved in the first type and conjugated B and T cell responses are involved in the last two types. Therefore, the ability to respond to T-dependent and T-independent antigens must be investigated under suspicion of B cell deficiency. Another important topic to be considered is age, as distinct immune responses can be observed in infants, adults and elderly individuals. As a rule of thumb, adequate antibody titers to some of these vaccines in children up to 15 months old indicate a normal humoral immune response.

#### Protein Antigens (Tetanus Toxoid, Diphtheria Toxoid, and Measles/Mumps Serologies)

Tetanus and diphtheria toxoids are the main targets of the antibody response against protein antigens frequently used for PAD assessment. Their potent immunogenicity associated with the classic worldwide-accepted three-dose immunization program (with an acellular or cellular *Bordetella pertussis* component, rubeolla and tetanus toxoid) given to infants by six months of age helps explain this preference.

Tetanus and diphtheria titers above 0.1 and up to 0.2 IU/mL, respectively, are considered protective, and seroconversion rates approach 100% one month after the second or third dose (60, 61). Moreover, low levels after a vaccine booster in adult patients who have not been vaccinated for several years are expected, but children who have recently received routine immunization are expected to present a prominent response (62). Therefore, immunization records are crucial for interpreting vaccine responses.

Several other vaccine protein antigens are suitable for IEI diagnosis proposal and are shown to present well-established protective levels. Vaccination with inactivated live virus, such as hepatitis A, polio (inactive) and influenza, or recombinant antigens, such as hepatitis B, is safer and recommended for all immunocompromised patients. In contrast, vaccines with viable antigens, such as measles, mumps, polio (oral) and rubella, made with attenuated viruses require more attention with respect to adverse outcomes. Postvaccine antibody responses to measles and mumps are also commonly employed in the investigation of IEI, and levels >1.1 enzyme international units/mL (EIU/mL) are considered adequate, presenting seroconversion rates of 95-99% and 100% after the first and second doses, respectively (60). Protective levels of antibodies against protein antigens are well established and controlled by CAP. For this topic, we recommend the remarkable reviews by Bonilla et al. in 2015 and 2020 (16, 60).

#### Polysaccharide Antigens (*Streptococcus pneumoniae*)

Due to their low immunogenicity, most polysaccharide vaccines are conjugated to a protein or glycoprotein carrier to enhance antibody production. Specific T-independent antibody responses are mostly assessed after administration of a 23-valent capsular unconjugated polysaccharide vaccine, which includes the 23 most prevalent serotypes (PPV-23) (63). The response to purified polysaccharide antigens is fully developed in 2-y/o children and the diagnosis of specific polysaccharide antibody deficiency (SPAD) must be considered in patients who receive PPV-23. If a previous immunization was performed with one of the conjugated vaccines, antibody titers for the other serotypes missing from the conjugated vaccine must be necessarily assessed, as protein conjugates induce antibodies indistinguishable from those induced by purified polysaccharides (62).

Serotype-specific IgG assessment by the well-standardized World Health Organization (WHO) ELISA is currently accepted as the ‘gold standard’ for the evaluation of antibody responses to pneumococcal serotypes (64), and this procedure recommends serum absorption with C-polysaccharide antigen and serotype 22 polysaccharide to remove nonprotective or cross-reactive antibodies. WHO ELISA has been shown to correlate closely with opsonophagocytosis assays (65), which is the only functional assay type available and is strongly correlated with vaccine efficacy. However, opsonophagocytosis assays are poorly used in clinical practice, as they have not been internationally standardized (66).

Antibody response against *S. pneumoniae* polysaccharide assessment is based on three main features: i) specific antibody levels increased over preimmunization levels; ii) the final concentration of antibodies after immunization; and iii) the percentage of serotypes against which an arbitrarily defined antibody titer was reached (67).

The protective serotype antibody level after protein-conjugated vaccines is not the same as that after PPV-23 vaccination. Protective levels are considered to be ≥0.35 μg/mL for each serotype after administration of protein-conjugated vaccines (68). After PPV-23 vaccination, children of 2 to 5 y/o are expected to develop at least a 2-fold increase in 50% of the serotypes tested, assuming that these levels are equal to or greater than 1.3 μg/mL (69). This cutoff level is considered protective against infection when assessed by ELISA, but the corresponding cutoff in multiplex immunoassay platforms has yet to be determined (66).

The multiplex addressable laser bead immunoassay (ALBIA) allows the simultaneous assessment of serum antibodies against the 23 pneumococcal polysaccharide serotypes present in PPV-23 (70–72). FDA-approved multiplex methods emerged in most reference laboratories as easier and faster attractive alternatives to ELISA that require significantly less sample volume, which is important in the pediatric population. Nonetheless, their correlations with WHO ELISA are variable, and significant result differences are reported by various laboratories (67, 73, 74).

### Isohemagglutinins

Isohemagglutinins or allohemagglutinins have been proposed as alternatives to the determination of the pneumococcal polysaccharide vaccine response, as they are clinically relevant and inexpensive indicators of the ability to mount an antipolysaccharide response (75). Isohemagglutinins comprise naturally occurring IgM and IgG anti-polysaccharide antibodies that cross react with erythrocyte surface antigens A and B, probably induced by contact with commensal gastrointestinal bacteria (76). Hence, these antibodies are usually undetected in newborns and patients with type AB blood. IgM and IgG isohemagglutinins can be ordered together or separately, and the results are semiquantitative and expressed in titers. Isohemagglutinin levels can be detected by 3-6 months of age, and 90% of adult titers are reached by 3 years of age, increasing to maximum levels between 5 and 10 years of age (77).

No cutoff values for isohemagglutinins are available. Most laboratories use a cutoff of 1/32 or 1/16. IEI specialists tend to use 1/8 for children below the age of 3 years and 1/16 for those above 3 years (78). Nevertheless, isohemagglutinins should not be used as a bona fide index of polysaccharide antibody response because different cutoff values (from 1/4 to 1/32) failed to discriminate individuals with SPAD from those with a normal Pn antibody response (78).

### B Cell Immunophenotyping (Flow Cytometry)

Defects in B cell development, selection or function lead to humoral immunodeficiencies. With multiple surface marker staining, multiparametric flow cytometry can identify B cell subsets in peripheral blood, which, in turn, can be useful for PAD diagnosis. In addition, dynamic changes in the B cell compartment composition are observed during aging and may also be addressed. Studies have reported that, similar to other lymphocyte populations, total B cell counts increase by 2-fold immediately after birth, remain high until 2 years of age, and thereafter gradually decrease by approximately 6.5-fold until adulthood (79). On the other hand, age-related modifications of B cell maturation as well as clinically unvalidated immunophenotyping panels represent challenges for standardization and quality control. Moreover, accuracy differences in monoclonal antibodies and individual and populational heterogeneity may also restrict reliable studies in this field.

Since the 1990s, a plethora of studies to standardize the total circulating B cell (CD19^+^ or CD20^+^) absolute count has been conducted within a wide age range in different populations (**Table 3**). Notably, an Italian national multicenter study defined reference ranges for normal values of CD19^+^ B cells in a large cohort of 1,311 healthy adults (blood donors and volunteers chosen according to the Italian law for donor selection) (91). Despite no significant differences observed between hematology counters and cytometers, some methodological variables represented inevitable causes of variability, such as the quantity of sample, washing protocols, monoclonal antibodies and instrument brands used. Similar results were recently reported in healthy children aged 4 months to 7 years, as a Han Chinese initiative was accomplished (114).

**Table 3:** Chronology of the main initiatives available in the medical literature for the standardization of total B cell circulating numbers, as rated according to the sample number, ethnicity and age range of recruited patients.

Authors	Year of publication	Sample (n)	Ethnicity	Age (y/o)	Ref.
Wiener et al.	1990	198	American	5-65	(80)
Reichert et al.	1991	271	Belgium, British, Swedish	18-70	(81)
Kotylo et al.	1993	130	American	0-17	(82)
Kontny et al.	1994	221	German	Newborns	(83)
Dhaliwal et al.	1995	152	Malay, Chinese and Indian	*	(84)
Roman et al.	1995	100	Romanian	Adults*	(85)
Kam et al.	1996	208	Chinese	18-71	(86)
Robinson et al.	1996	233	British	5-13	(87)
Comans-Bitter et al.	1997	429	Deutch	0-16	(88)
Huppert et al.	1998	513	British	64- >80*	(89)
Shahabuddin et al.	1998	132	Saudi Arabian	0-13; 18-44	(90)
Santagostino et al.	1999	1311	Italian	18-70	(91)
Al Qouzi et al.	2002	209	Saudi Arabian (male)	18-44	(92)
Kaaba et al.	2002	127	Kuwaiti Arab	18-59	(93)
Swaminathan et al.	2003	138	Indian	3-15	(94)
Shearer et al.	2003	807	American	0-18	(95)
Ikincioğullari et al.	2004	190	Turkish	0-18	(96)
Timová et al.	2004	495	Central and Eastern European	9-11	(97)
Chng et al.	2004	232	Singaporean (Chinese, Malay, Indian, Caucasian and Eurasian)	16-65	(98)
Bisset et al.	2004	70	Swiss	Adults*	(99)
Yaman et al.	2005	220	Turkish	18-80	(100)
Jentsch-Ullrich et al.	2005	100	German	19-85	(101)
Das Gupta A, Ochani Z	2006	185	Indian	18-49	(102)
Branch et al.	2006	112	Afro-Caribbean	Adults*	(103)
Al-Jabri et al.	2008	118	Omani (male)	18-51	(104)
Jiao et al.	2009	151	Chinese	19-83	(105)
Shoormasti et al.	2011	233	Iranian	20-45	(106)
Wong et al.	2013	273	Hong Kong Chinese	17-59	(107)
Al-Mawali et al.	2013	50	Omani	18-57	(108)
Kamallou et al.	2014	221	Iranian	20-40	(109)
Choi et al.	2014	294	Korean	21-80	(110)
Valiathan et al.	2014	150	American	12-18; 21-67	(111)
Valdiglesias et al.	2015	144	Spanish	65-95	(112)
Al-Thani et al.	2015	150	Qatari	18-55	(113)
Jia et al.	2015	1027	Han Chinese	0-7	(114)
Shahal-Zimra et al.	2016	326	Israeli	17-94	(115)
Qin et al.	2016	1068	Chinese	18-80	(116)
Azarsiz et al.	2017	90	Turkish	0-18	(117)
Kokuina et al.	2019	129	Cuban	18-80	(118)
El Allam et al.	2020	83	Moroccan	0-18	(119)
Lerkvaleekul et al.	2020	182	Thai	0-15	(120)

*Exact data not available.

In addition, as total circulating B cell assessment was rapidly popularized in immunology diagnostic labs around the world, quality control programs were needed to determine intra- and interlaboratory coefficients of variation, standardize procedures, define the best blood tubes and anticoagulants and, therefore, ensure comparable results, which was an issue predicted by studies in the late 1980s (121–124). The first robust initiative was conducted in 1990 with 3-year interlaboratory proficiency testing for lymphocyte subset phenotyping, consisting of part of the French Etalonorme national quality control program (125). The authors concluded that calibration standards and instrument calibration procedures affect average cell counts; hence, the inclusion of lyophilized cells in each evaluation would offer a longitudinal approach for evaluating intra- and interlaboratory results. In 2000, the Belgian Scientific Institute of Public Health introduced a voluntary external quality assessment scheme for lymphocyte immunophenotyping, including CD19^+^ B cells, and demonstrated median intralaboratory coefficients of variation in cell percentages and absolute numbers of 3.2% and 16.5%, respectively (126). Although the topic was not discussed by the Belgian authors, one can argue that the higher absolute number intralaboratory variation observed may be caused by cell blood count variability, but this is an important bias to be solved. Later, a 10-year experience of expanded quality control study englobing all Benelux countries was published, and assay variability tended to decline with time (127). Currently, CAP offers quality management programs by sending standard samples worldwide to voluntary participating labs and monitoring progress over time.

On the other hand, studies for the standardization and quality control of circulating B cell subsets are not as widespread as those for the total B cell count. Using CD27 as a surrogate marker of human memory B cells and CD38, immunoglobulin (Ig) M and IgD as differentiation markers, B cells have been divided into five different populations according to their differentiation stage in the lymphoid organs (128): naïve B cells (CD27^-^ IgD^+^); nonswitched memory B cells (CD27^+^ IgD^+^ IgM^+^); classical switched memory B cells (CD27^+^ IgD^-^ IgM^-^); transitional B cells (CD38^high^ IgM^high^); and plasma cells (CD38^high^ IgM^-^). In addition, a CD21^low^ CD38^low^ B cell subset has been previously shown to be expanded in autoimmune diseases and immunodeficiencies (129, 130). Thus, due to its simplicity, this 5-marker immunophenotyping panel (CD27, IgM, IgD, CD38 and CD21) has been commonly used to assess peripheral B cell maturation, and some standardization initiatives have already been conducted, although no quality control proposal is available to the best of our knowledge.

Based on this panel, Piatosa et al. (131) determined reference values for B cell subsets in healthy Polish children. Simultaneously, Morbach et al. (132) also established age-dependent reference values for distinct peripheral blood B cell populations in a cohort of individuals ranging from neonates to adults using the same immunophenotyping panel. Kverneland et al. (133) and Garcia-Prat et al. (134) determined reference values in adult Caucasian individuals older than 20 years and a pediatric Spanish population under 18 years, respectively. Although similar to that used by Piatosa et al. (131) and Morbach et al. (132), the immunophenotyping panel used by Kverneland et al. (133) and Garcia-Prat et al. (134) presented slight differences, including CD38^dim^ for class-switched and nonswitched memory B cells and CD24 expression for transitional cell and plasmablast assessment. Similarly, the EuroFlow PID group added two additional surface markers (CD5 and CD24) and conducted a comprehensive study addressing the distribution of normal B cell subsets in a wide age range: from cord blood to >80 y/o subjects (79). The EuroFlow staining strategy further subclassified memory B cells and plasma cells according to their membrane immunoglobulin isotype (IgG subclasses, IgA_1_ and IgA_2_). At first sight, these slight modifications seem innocuous; however, they are enough to impede comparison with previously cited studies.

### Intracellular BTK Expression

X-linked agammaglobulinemia (XLA) is the most common form of inherited agammaglobulinemia, comprising 70% of all cases, and is caused by mutations in a pivotal protein for early pre-B cell receptor intracellular signaling: Bruton’s tyrosine kinase (BTK). As XLA patients lack B cells, the deficient expression of mutated BTK can be assessed by flow cytometry using monocytes and platelets (135). Interestingly, this method is useful for the detection of not only XLA, but also BTK-deficient female carriers (136).

Although helpful for XLA diagnosis, monoclonal antibody standardization and optimal diagnostic cutoff values of intracellular BTK expression have yet to be determined, in turn requiring a simultaneous healthy control sample in every test. In addition, to our knowledge no reference range or interlaboratory quality assessment protocols have been determined.

### Defective Cell Surface CVID-Related Protein Expression

CVID is the most common symptomatic PAD in adults, and diagnosis is mainly guided by clinical history, low immunoglobulin serum levels, defective vaccine responses and typical B cell immunophenotyping results. Approximately 30% of these cases may have an underlying genetic etiology, which, in turn, can be additionally confirmed by a flow cytometry-based assessment of the causative protein. At least 27 CVID-related monogenic conditions have been identified to date. Interestingly, other cases carry variants of undetermined significance that can be validated by the same approach. Although specific CVID-causative mutated proteins represent only a fraction of all patients, some can be addressed by flow cytometry, such as TACI (137), BAFF-R (138), ICOS (139), CD19 (138), CD21 (140, 141), and ICOSL (142), among others. Nevertheless, these assays are not simple, as most require stimulation of the cells, and a few are seldom useful, for example, TACI (TNFRSF13b).

To date, we were not able to find any initiative for standardization or diagnostic accuracy assessment of such cell CVID-related surface protein expression. In addition, reference intervals and interlaboratory quality control programs for these methodologies have yet to be established. Hence, most labs recommend comparison with a simultaneously analyzed unrelated healthy control sample.

## Combined Immunodeficiencies or Immunodeficiencies Affecting Cellular and Humoral Immunity

SCID comprises a group of rare, monogenic disorders characterized by a blockade of the development of lymphoid stem cells into pre-T cells, with or without abnormal B and/or natural killer (NK) cell differentiation. Recently, several molecular defects causing SCID have been identified along with many other conditions causing incomplete T cell immunodeficiencies, which, in turn, are referred to as atypical SCID or, simply, combined immunodeficiencies (CID). This group of diseases presents early clinical manifestations with a spectral history of failure to thrive, unexplained diarrhea, interstitial pneumonitis, hepatosplenomegaly, oral candidiasis and other recurrent bacterial, viral, fungal or protozoal infections. The recent strategies comprising both early newborn screening and accurate diagnosis with lab tests detailed below allowed significant improvement in the proper specialized treatment and life expectancy of these patients (143, 144).

### T Cell Receptor Excision Circles

Quantification of the copy number of T cell receptor excision circles (TREC) in peripheral blood, which is usually performed by quantitative real-time polymerase chain reaction (qRT-PCR), has been shown to be an effective tool for the early identification of severe T lymphocyte deficiencies. Quantitative analysis of TREC is frequently used to: i) estimate the thymopoiesis rate in newborn screening tests for SCID (145–147); ii) assess thymus involvement in autoimmune diseases (148, 149); and iii) evaluate T cell reconstitution during acquired immunodeficiency syndrome antiretroviral therapy and after bone marrow transplantation (150–154).

The TREC quantitative assay, initially proposed by Douek et al. (152) has been modified in different ways, which hampers result comparisons among different approaches. Newborn TREC quantification, which is performed using DNA extracted from dried blood spots, is a simple, low-cost methodology and maintains sample stability, making it an ideal collection strategy (155). However, there are divergences among the different assays and units used to measure TREC, impeding interpretation and comparison among data. Values are often expressed as the absolute number of TREC molecules per μg of DNA within peripheral blood mononuclear cells or T lymphocytes or per 10^6^ cells as an extrapolation of the recovery of 1 μg of DNA from approximately 150,000 cells (156). Another important issue is that there are no well-established age-specific reference intervals for SCID diagnosis, since most patients are infants and young children. Normally, an initial cutoff value for TREC quantification is used to determine whether a sample is within the normal range. Samples with TREC levels below the cutoff are usually sent for confirmatory tests (immunophenotyping of T cell subpopulations and genetic analyses). Each laboratory has established its own cutoff, as previous studies used a wide number of samples and advocated a screening sensitive cutoff of 25 copies/μL, below which further clinical and laboratory investigation is required (157–159). Notably, the absence of a global reference range is not an issue, but a thorough standardization process in each lab is absolutely recommended. We suggest that a single cutoff may not be as representative as local reference intervals in healthy individuals of different age groups (145, 160). Therefore, it is important to establish cutoff values for assumed positive results based on assays using a sufficient number of samples (normal and diagnosed SCID cases) prior to test implementation as part of neonatal screening programs to avoid unnecessary patient recall.

### T Cell Immunophenotyping

As HIV spread worldwide in late 1980s and CD4/CD8 T cell assessment proved useful in the management of AIDS patients, innumerous studies attempted to determine the reference range of total T cells and helper/cytotoxic subsets among different populations. **Table 4** summarizes the main initiatives to date to the best of our knowledge, albeit a comprehensive review of this topic would require an exclusive chapter. Simultaneously, external quality control and interlaboratory reproducibility assessment approaches were demanded during the 1990s, resulting in the organization of different national groups. One of the largest initiatives in the area was headed by the National Institute of Allergy and Infectious Diseases Division of AIDS (NIAID-DAIDS) (204), which, since 1999, has funded the Immunology Quality Assessment Program with the goal of assessing proficiency in basic lymphocyte subset immunophenotyping for each North American laboratory (205, 206). Nevertheless, other groups with similar purposes had previously succeeded not only in the United States (207) but also in Bulgary (208), Italy (209) and the United Kingdom (210). Unsurprisingly, further initiatives developed afterward in Europe (126, 127), Africa (211–213), Asia (214, 215) and South America (216).

**Table 4:** Chronology of the main initiatives available in the medical literature for the standardization of circulating T cell numbers and CD4/CD8 subsets, as rated according to the sample number, ethnicity and age range of recruited patients.

Authors	Year of publication	Sample (n)	Cell population	Ethnicity	Age (y/o)	Ref.
Denny et al.	1992	208	CD3/CD4/CD8	American	1-5	(161)
Kotylo et al.	1993	130	CD3/CD4/CD8	American	0-17	(82)
Howard et al.	1996	215	CD3/CD4/CD8	American	18-67	(162)
Comans-Bitter et al.	1997	429	CD3/CD4/CD8	Deutch	0-16	(88)
Lisse et al.	1997	803	CD4/CD8	Bissau-Guinean	0-6	(163)
Shahabuddin et al.	1998	132	CD3/CD4/CD8	Saudi Arabian	0-13; 18-44	(90)
Tsegaye et al.	1999	485	CD3/CD4/CD8	Ethiopian	15-45	(164)
Al Qouzi et al.	2002	209	CD3/CD4/CD8	Saudi Arabian (male)	18-44	(92)
Swaminathan et al.	2003	138	CD3/CD4/CD8	Indian	3-15	(94)
Shearer et al.	2003	807	CD3/CD4/CD8	American	0-18	(95)
Uppal et al.	2003	94	CD4/CD8	Indian	18-74	(165)
Chng et al.	2004	232	CD3/CD4/CD8	Singaporean (Chinese, Malay, Indian, Caucasian and Eurasian)	16-65	(98)
Bisset et al.	2004	70	CD3/CD4/CD8	Swiss	Adults*	(99)
Lugada et al.	2004	3311	CD3/CD4/CD8	Ugandan	0-92	(166)
Bussmann et al.	2004	437	CD4/CD8	Botswanan	19-36	(167)
Jiang et al.	2004	614	CD4/CD8	Chinese	16-50	(168)
Amatya et al.	2004	200	CD3/CD4/CD8	Indian	18-55	(169)
Gomo et al.	2004	1113	CD4/CD8	Zimbabweans (pregnant)	Adults*	(170)
Yaman et al.	2005	220	CD3/CD4/CD8	Turkish	18-80	(100)
Jentsch-Ullrich et al.	2005	100	CD3/CD4/CD8	German	19-85	(101)
Aina et al.	2005	864	CD4	Nigerian	10-69	(171)
Ampofo et al.	2006	249	CD4/CD8	Ghanaian	18-83	(172)
Klose et al.	2007	186	CD4/CD8	Burkinabe	18-78	(173)
Al-Jabri et al.	2008	118	CD3/CD4/CD8	Omani	18-57	(104)
Das et al.	2008	252	CD3/CD4/CD8	Indian	Adults*	(174)
Ngowi et al.	2009	102	CD4/CD8	Tanzanian	Adults*	(175)
Murugavel et al.	2009	213	CD3/CD4/CD8	Indian	Adults*	(176)
Chama et al.	2009	541	CD4	Nigerian	Adults*	(177)
Oladepo et al.	2009	2570	CD4/CD8	Nigerian	18- >60*	(178)
Lawrie et al.	2009	678	CD4	South African	*	(179)
Buchanan et al.	2010	655	CD4/CD8	Tanzanian	0-18	(180)
Shoormasti et al.	2011	233	CD3/CD4/CD8	Iranian	20-45	(106)
Sagnia et al.	2011	352	CD3/CD4/CD8	Cameroonian	0-6	(181)
Thakar et al.	2011	1206	CD3/CD4	Indian	17-72	(182)
Pennap et al.	2011	444	CD4	Nigerian	15-44	(183)
Adoga et al.	2012	1123	CD3/CD4	Nigerian	0-50	(184)
Shakya et al.	2012	602	CD3/CD4/CD8	Nepalese	18-60	(185)
García-Dabrio et al.	2012	319	CD3/CD4/CD8	Spanish	4-88	(186)
Touil et al.	2012	242	CD3/CD4/CD8	Moroccan	19-49	(187)
Wong et al.	2013	273	CD3/CD4/CD8	Hong Kong Chinese	17-59	(107)
Al-Mawali et al.	2013	50	CD3/CD4/CD8	Omani	18-57	(108)
Moreno-Galván et al.	2013	400	CD3/CD4/CD8	Mexican	20-40	(188)
Torres et al.	2013	925	CD3/CD4/CD8	Brazilian	2-6; 19-56	(189)
Kamallou et al.	2014	221	CD3/CD4/CD8	Iranian	20-40	(109)
Valiathan et al.	2014	150	CD3/CD4/CD8	American	12-18; 21-67	(111)
Atanasova et al.	2014	72	CD3/CD4/CD8	Bulgarian	Newborns	(190)
Tembe et al.	2014	257	CD3/CD4/CD8	Mozambican	18-24	(191)
Jia et al.	2015	1027	CD3/CD4/CD8	Han Chinese	0-7	(114)
Al-Thani et al.	2015	150	CD3/CD4/CD8	Qatari	18-55	(113)
Prasetyo et al.	2015	241	CD4	Javanese	18-65	(192)
Shahal-Zimra et al.	2016	326	CD3/CD4/CD8	Israeli	17-94	(115)
Qin et al.	2016	1068	CD3/CD4/CD8	Chinese	18-80	(116)
Zhang et al.	2016	268	CD3/CD4/CD8	Chinese	21-60	(193)
Afolabi et al.	2017	1205	CD4	Nigerian	0-65	(194)
Mulu et al.	2017	481	CD4	Ethiopian	18-65	(195)
Yeshanew et al.	2017	400	CD3/CD4/CD8	Ethiopian (pregnant)	18-40	(196)
Genetu et al.	2017	200	CD4	Ethiopian (pregnant)	18-42	(197)
Enawgaw et al.	2018	967	CD4	Ethiopian	18-61	(198)
Karn et al.	2018	207	CD3/CD4	Nepalese	0-14	(199)
Kokuina et al.	2019	129	CD3/CD4/CD8	Cuban	18-80	(118)
Louati et al.	2019	143	CD3/CD4/CD8	Tunisian	18- >45*	(200)
Mishra et al.	2020	400	CD3/CD4	Nepalese	15-60	(201)
Niu et al.	2020	150	CD4	Han Chinese	20-70	(202)
Scheffer-Mendoza et al.	2020	50	CD3/CD4/CD8	Mexican	Newborns	(203)

*Exact data not available.

The steps in T cell maturation process are regulated by a complex transcriptional network, which mediates the homing, proliferation, survival, and differentiation of developing T cells (217–219).Therefore, unique combinations of surface markers can identify different T cell subsets with distinct functions (220). In clinical practice, a CD45RA^+^/CD45RO^+^ imbalance toward memory T cells in a phenotypically suspected child may drive the diagnosis of combined immunodeficiencies. To the best of our knowledge, the first study aiming to standardize CD45RA^+^ T cells dates to 1992 in Spain (221). Other studies aiming to standardize the phenotyping of CD45RA^+^ naïve and CD45RO^+^ memory T cells have since been conducted in Kuwaiti Arabian (93), American (222), German (223), Italian (224), Dutch (225, 226), Brazilian (227) and Moroccan (228) healthy donors of different ages.

Although CD45RA, CD45RO, CD62L and C-C chemokine receptor 7 (CCR7) are the most common markers used for T cell maturation immunophenotyping indicated for CID diagnosis, the existence of several additional markers may result in challenging heterogeneity in laboratory reports from different services (218). Therefore, a consensus on the phenotypic definition of the various T cell subsets should be established, which will pave the way for robust standardization studies. Currently, different combinations of markers used to define such cells complicate comparability between studies and laboratories worldwide. **Table 5** shows standardization studies using mainly CD45RA/CD45RO/CCR7/CD62L-derived T cell subsets and, moreover, exemplifies the striking heterogeneity of immunophenotyping panels. Qin et al. (116) and Shearer et al. (95) determined the absolute number and percentage of T cell subsets using similar markers in the largest cohorts of adult and pediatric populations, respectively. Interestingly, the authors additionally determined the frequency of activation-primed (CD28^+^) and activated (HLA-DR^+^/CD38^+^) helper and cytotoxic T cells.

**Table 5:** Chronology of the main initiatives available in the medical literature for the standardization of circulating naïve and memory T cell subsets, as rated according to the sample number, ethnicity and age range of recruited patients.

Authors	Year of publication	Sample (n)	T cell subset markers	Ethnicity	Age (y/o)	Ref.
Shearer et al.	2003	807	CD45RA CD45RO CD62L HLA-DR CD38	American	0-18	(95)
Bisset et al.	2004	70	CD45RA CD45RO CD62L HLA-DR CD38	Swiss	Adults*	(99)
Jiao et al.	2009	151	CD45RA CD45RO CD62L HLA-DR CD28 CD38	Chinese	19-83	(105)
Sagnia et al.	2011	352	CD45RA CD45RO CD62L HLA-DR CD38	Cameroonian	0-6	(181)
Moraes-Pinto et al.	2014	463	CD45RA CCR7 CD38 CD27	Brazilian	0-48	(229)
Valiathan et al.	2014	150	CD45RA CD45RO CD62L HLA-DR CD28 CD38	American	12-18; 21-67	(111)
Bretschneider et al.	2014	66	CD45RA CCR7 CD27 CD57	German	0-72	(230)
Qin et al.	2016	1068	CD45RA CD45RO CD62L HLA-DR CD28 CD38	Chinese	18-80	(116)
Garcia-Prat et al.	2019	159	CD45RA CD45RO CCR7	Spanish	0-18	(134)

The immunophenotyping panel used by each paper is also depicted, which consisted of different combinations of the staining markers CD45RA, CD45RO, CCR7, CD62L and HLA-DR.

*Exact data not available.

The multicentered EuroFlow and PERISCOPE (PERtussIS COrrelates of Protection Europe) consortia recently validated a 14-color immune monitoring flow cytometric tube capable of distinguishing more than 89 CD4^+^ T cell populations in peripheral blood, including several maturation and differentiation stages during aging, in 145 healthy donors (231). Unfortunately, despite comprehensive charts, no specific reference range was reported. A CAP quality assessment program is available for credited labs that voluntarily accept receiving regular heparinized whole blood samples to quantify CD45RA^+^ naïve, recent thymic emigrant (CD45RA^+^ CD31^+^), CD45RO^+^ memory and terminally differentiated effector memory (CD8^+^ CD45RA^+^ CCR7^-^) T cells. In addition to the low number of predefined T cell subsets, this strategy is also limited due to complications of cell viability in long-distance shipment and result comparability. Finally, to the best of our knowledge, no other quality assessment proposal is available regarding a broader T cell immunophenotyping panel.

### Th17 Immunophenotyping

Th17 differentiation is mainly mediated by intracellular STAT3 activation. Therefore, STAT3 loss-of-function (LOF) or gain-of-function (GOF) mutations may equally impair circulating Th17 cell numbers in autosomal dominant hyper-IgE syndrome or autoimmune disease, multisystem, infantile-onset 1 (232). Despite the apparent usefulness of assessing Th17 cell numbers by flow cytometry for diagnostic purposes, a standard immunophenotyping panel has yet to be defined. Moreover, a validated reference range for circulating Th17 cell numbers is usually unavailable, which makes running a simultaneous healthy control sample mandatory for result comparison. Botafogo et al. (231) recently analyzed 113 samples from healthy controls aged 0-89 years to establish reference values for Th17 cells defined as CD183^–^/CD194^+^/CD196^+^/CCR10^–^. According to the authors, these cell surface markers were proven accurate in identifying IL17A-producing cells. Similarly, Niu et al. (202) established distributions and reference ranges for stimulated CD4^+^ IL17-producing cells in 150 healthy Chinese healthy volunteers aged 20-70 years. However, we were not able to find more data regarding Th17 cell reference ranges in other populations. In addition, no quality assessment program for Th17 immunophenotyping is available.

### Intracellular Wiskott-Aldrich Protein Expression

**Figure 1:**
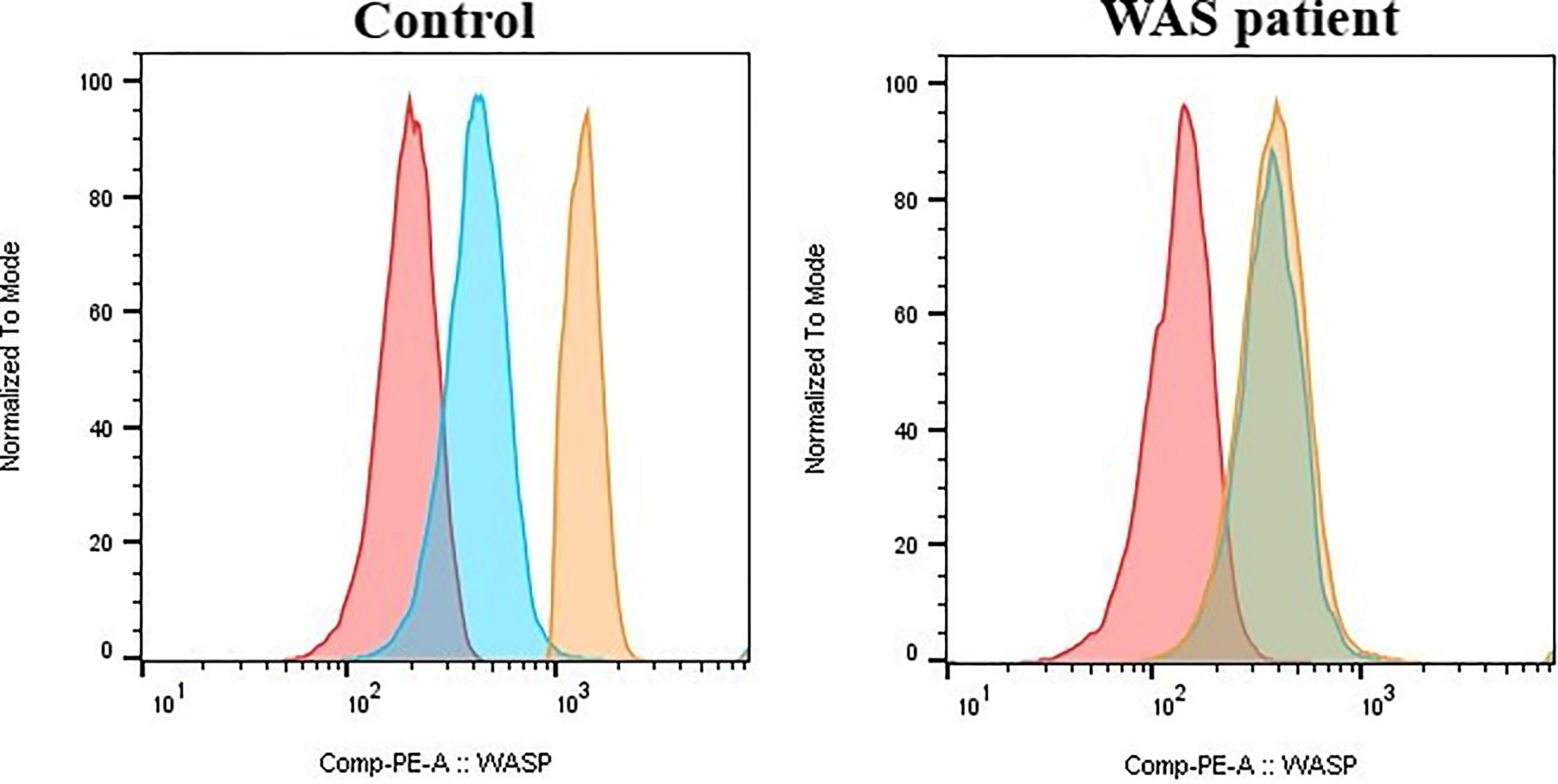
Wiskott-Aldrich (WAS) intracellular protein expression in gated lymphocytes determined by flow cytometry. The median fluorescence intensity is significantly reduced in WAS patients carrying the truncated protein. red: unstained; blue: immunoglobulin isotype control; orange: Wiskott-Aldrich protein.

Flow cytometry-based assessment of intracellular WAS protein (WASP) is useful for screening patients suspected to have WAS or X-linked thrombocytopenia and neutropenia (233) and for following up chimerism after hematopoietic stem cell transplantation or somatic reversion mosaicism (234). Despite its use in immunology clinics worldwide, methodology standardization, diagnostic accuracy and optimal diagnostic cutoff values for flow cytometric WASP measurement are still lacking. Similarly, reference intervals and interlaboratory quality assessment programs for intracellular WASP expression have not been determined. Therefore, a simultaneous healthy control sample run is recommended to validate results (**Figure 1**). Recently, Rawat et al. (235) suggested a stain index ratio using the median fluorescence intensities of patients and controls and found that values lower than 0.65 for gated lymphocytes are suggestive of WAS. Regardless, a broader validation of other centers is still needed.

### Defective Cell Surface or Intracellular SCID-Related Protein Expression

SCID diagnosis is mainly guided by clinical history, newborn TREC screening, typical T cell immunophenotyping results and potentially impaired lymphoproliferation in response to mitogens. Once a diagnosis is made, gene sequencing analyses may determine the underlying etiology, which can also be confirmed by a flow cytometry-based assessment of the defective protein. On the other hand, although approximately 2030% of those cases remain without any identifiable pathogenic mutation (236, 237), some carry variants of undetermined significance, which, in turn, can be validated by appropriate assays.

More than 50 SCID-causative molecular targets have been identified to date. Similar to specific PAD-causative proteins, some molecules are qualified to be addressed by a flow cytometric CID and SCID-driven diagnostic approaches, namely, CD132 (IL-2Rγ) (238), CD127 (IL-7Rα) (239), major histocompatibility complex I (240) and II (241), CD45 (242), CD3 chains (239), DOCK8 (243), and IKAROS (244).

Despite our lack of intention to exhaust this topic and the natural difficulty of validating a methodology for an uncommon condition with even rarer subtypes, we are not aware of available protocols for test standardization and quality assessment programs thus far. Moreover, reference intervals for defective cell surface and intracellular SCID-related protein expression are still lacking. Therefore, for the analytes discussed above, a simultaneous healthy control sample run is pivotal for comparison.

### Adenosine Deaminase and Purine Nucleoside Phosphorylase Activity

Adenosine deaminase 1 (ADA-1) deficiency is an autosomal recessive disorder resulting in a heterogeneous form of combined immunodeficiency. Specific diagnosis of ADA-1 deficiency in immunodeficient patients can be achieved by enzyme activity or metabolite quantification assays of several easily available cell types, usually erythrocytes. Affected individuals have less than 1% normal ADA-1 catalytic activity in red cell hemolysates. Kinetic ADA activity assays have been extensively reproduced since their initial description (245–250), allowing companies to develop fluorometric and spectrophotometric assays. Despite commercially available standardized tests, diagnostic accuracies and reference ranges remain unestablished. This may partially explain several reports of ADA-1-deficient patients without immunodeficiency (251, 252). Of note, to the best of our knowledge, only two small uncontrolled studies have systematically determined ADA-1 erythrocyte activity in healthy controls (253, 254). No specific quality assessment program for ADA-1 erythrocyte activity has been proposed to date.

Similar to ADA-1, purine nucleoside phosphorylase (PNP) participates in the purine salvage pathway. PNP deficiency can result in a rare CID with associated syndromic features. Low PNP activity in erythrocyte lysates can be assessed by liquid chromatography-tandem mass spectrometry and enzymatic colorimetric assay (255, 256). However, to our knowledge, no study determining reference values in a healthy control population or establishing a quality assessment has been found.

## Diseases of Immune Dysregulation

Approximately 30% of all monogenic IEI described thus far have a clinical phenotype predominantly resulting from a maladaptive change in molecular control leading to immune regulation breakdown, such as autoimmunity, autoinflammation, lymphoproliferation, malignancy and severe atopy, rather than infections (1). This group of disorders is rapidly growing and has been recently termed primary immune regulatory disorders (PIRD) (1, 257). Despite limited importance, routine nonfunctional immunology labs can be helpful under specific situations, as follows.

### Hemophagocytic Lymphohystiocytosis

Hemophagocytic lymphohistiocytosis (HLH) is a life‐threatening hyperinflammatory disease mainly in children younger than 1 year manifested by high persistent fever, pancytopenia, hepatosplenomegaly, and elevated aminotransferase and ferritin levels (258).The cytotoxic activity of CD8^+^ T cells and natural killer (NK) cells is impaired in primary HLH, impeding the elimination of virus‐infected cells and instead causing continuous secretion of inflammatory cytokines, especially soluble CD25.

#### Soluble CD25

CD25 is strongly expressed after T cell activation. Upon activation, a 40-45-kD truncated protein is cleaved off of the 55-kD IL-2Rα membrane protein and shed into circulation as the soluble IL-2 receptor (sIL-2R or sCD25) (259). Thus, sCD25 is considered a surrogate marker of T cell activation, and elevated serum levels have been described in various diseases, including hematological malignancies (e.g., HTLV-1-associated T cell leukemia, hairy cell leukemia, acute lymphoblastic leukemia, and non-Hodgkin lymphoma), infections (e.g., human immunodeficiency virus, viral hepatitis, and Epstein-Barr virus), autoimmune conditions (e.g., rheumatoid arthritis, sarcoidosis, systemic lupus erythematosus, systemic juvenile idiopathic arthritis, Kawasaki’s disease, and autoimmune lymphoproliferative syndrome/ALPS), allograft rejection and graft−vs.−host disease after allogeneic hematopoietic stem cell transplantation (260, 261). In addition, sCD25 is also released from dendritic cells, activated B cells, monocytes, and malignant cells (260). sCD25 levels have been incorporated as one of the eight laboratory and clinical criteria for HLH diagnosis, of which five must be met for diagnosis.

The currently available methods for sCD25 assessment are ELISA, whose results are expressed as pg/mL, and chemiluminescent immunoassay (ChLIA), with results expressed as U/mL. Although these assays present good correlation, the differing units may cause confusion. The cutoff has been defined as 2400 U/mL for pediatric patients, which may correspond to approximately 20,000 pg/mL in ELISA. A similar cutoff has been defined for adults (262), although it has been described that ELISA-determined sCD25 levels are higher in children (age 1-14 years) and elderly individuals (age 67-99 years) than in adults (age 22-67) (263, 264). The normal range in adults was set as 241-846 U/mL (265).

Damoiseaux et al. (259) reported a ChLIA sCD25 cutoff of 600 U/mL, which is equivalent to an ELISA cutoff between 4200 and 4800 pg/mL. Most clinical laboratories have set ELISA cutoffs between 2500 and 3500 pg/mL, although different strategies for sCD25 serum level cutoff standardization are adopted, generally based on the mean plus two standard deviations. Repeated sCD25 serum level assessment is also helpful for treatment monitoring and prognostic risk scoring in several conditions (261).

#### Intracellular Protein Expression – PRF1, SAP/SH2DIA, XIAP

(266)Some forms of primary HLH present defects that can be assessed by flow cytometry (267). One of these targets is perforin, which is easily quantified by intracellular staining flow cytometry; moreover, defects in granule transport can be screened by CD107a (LAMP1) exocytosis evaluated by flow cytometry (268, 269), as well as X-linked lymphoproliferative (XLP) analysis (270, 271). Once again, despite our lack of intention to exhaust this topic and the natural difficulty of validating a methodology for an uncommon condition, to the best of our knowledge, no protocols for test standardization and quality assessment programs are available thus far.

### ALPS

Autoimmune lymphoproliferative syndrome (ALPS) is a group of human disorders caused by genetic defects disrupting lymphocyte apoptosis (272). Currently, this expanding group of disorders includes prototypical autoimmune lymphoproliferative syndrome (ALPS, OMIM #601859), which is caused by defects in the FAS pathway of apoptosis (*FAS*, *FASLG*, and *CASP10*), and RAS-associated autoimmune leukoproliferative disorder (RALD), which is caused by somatic mutations in *NRAS* or *KRAS*. Most patients harbor pathogenic variants in the *FAS* gene inherited in an autosomal dominant fashion (272). Typical clinical findings include benign, chronic lymphadenopathy and splenomegaly; autoimmune cytopenias; and a high risk for lymphoma development (273). The classical laboratory hallmark of ALPS is the presence of circulating mature α/β receptor-carrying T cells that do not express CD4 or CD8 (double-negative T cells), which is a finding required for diagnosis (274). Other common laboratory manifestations include hypergammaglobulinemia, the presence of autoantibodies directed to blood cell elements, high levels of vitamin B12 and increase in soluble cytokines such as IL-10, IL-18 and soluble Fas ligand.

#### Double-Negative TCRα/β Circulating T Cells (DNT)

As a hallmark and required diagnostic finding in ALPS, the measurement of circulating double-negative T cells (DNT) is performed by flow cytometry (275, 276). This assay is easily conducted with a four-color instrument, and standardization requires running a panel of normal individuals to define the normal range in a particular laboratory, as is common practice for other flow cytometry assays. Gating was performed using T cell receptor (TCR) α/β, CD3, CD4 and CD8 staining. Values of CD3^+^ TCRαβ^+^ CD4^−^ CD8^−^ DNT cells above ≥ 1.5% of total lymphocytes or 2.5% of CD3^+^ lymphocytes in the setting of normal or elevated lymphocyte counts are considered abnormal, but these values may vary slightly among particular laboratories (274). Once established, the assay can undergo external quality assessment by interlaboratory exchanges, as many labs around the country perform the assay. There are no commercially available CAP controls for this measurement.

#### Soluble Mediators: IL-10, IL-18, Soluble FASL and Vitamin B12

The elevation of soluble cytokines and vitamin B12 was noted in ALPS patients early, particularly in those with *FAS* mutations (277, 278). These levels were later systematically measured in a large cohort of patients and controls and noted to have high positive and negative predictive values for the presence of *FAS* mutations (278). In particular, the combination of high DNT cells with elevated soluble FASL was shown to be a very potent predictor of *FAS* LOF mutations (278). Measurements of IL-10, IL-18 and sFASL can easily be performed by ELISA or ALBIA or similar protein immunoassays. A panel of controls should be run to define the range of normal values, and external quality assessment can be performed by interlaboratory sample exchanges.

### IPEX

IPEX syndrome is a rare monogenic primary immunodeficiency caused by *FOXP3* LOF mutations, which encodes a pivotal transcription factor required for the development of regulatory T cells. Treg cell absence or dysfunction are the main pathogenic events associated with early onset multiorgan autoimmunity in IPEX. We will discuss the main findings on standardization and quality assessment for circulating Treg cell numbers.

#### T Regulatory Cell Number

Several immunophenotyping panels have been suggested to discriminate circulating Treg cells. Despite controversies regarding the most appropriate panel, the literature has lately converged to a 4-marker panel: CD4^+^/CD25^+^/CD127^low^/Foxp3^+^. A consensus on the immunophenotyping definition based on 40 European and American experts was recently proposed and included a robust gating strategy for the context-dependent analysis of Tregs by flow cytometry (279). Later, a French initiative provided a perspective on methodological standardization and analysis using human Treg data obtained from healthy donors, transplanted patients, and, furthermore, parallel standard murine strains (C57BL/6 and BALB/c) (280). Recent studies also standardized the flow cytometry procedure for monitoring Treg cells stained with the CD4^+^/CD25^+^/CD127^low^/Foxp3^+^ panel associated with other markers (281, 282).

Regarding normal range standardization, Kim et al. (283) established reference intervals for CD4^+^/CD25^high^/Foxp3^+^ Treg cells in umbilical cord blood from 120 healthy neonates, highlighting that Treg cell numbers are higher in newborns, particularly in premature infants (284). Moreover, Niu et al. (202) recently determined the reference ranges of circulating Treg cells in 150 gender-balanced healthy adults of the Han Chinese population aged 20-70 years. Nevertheless, to the best of our knowledge, no additional data are available regarding Treg cell reference ranges in other populations or quality assessment programs. Therefore, a concurrent healthy control sample run is mandatory to determine whether Foxp3 expression is comparable.

## Defects in Phagocytes, and Intrinsic and Innate Immunity

The main feature of the innate immune system relies upon a limited germline repertoire of alarmins and receptors for common biochemical signature detection of danger and invading pathogens. Innate immunity receptor-induced intracellular signaling and cell activation are not restricted to the immune system, but also include nonhematopoietic cells. Therefore, defects in intrinsic and innate immunity encompass a heterogeneous group of disorders with systemic susceptibility to specific categories of infectious agents, such as mycobacteria, invasive pyogenic bacteria, viruses, parasites, and fungi. On the other hand, congenital impairment of phagocytes, as the main innate immunity effector cells, is associated with a similar clinical phenotype. Some monogenic conditions encoding truncated proteins classified within these two groups of diseases may be identified by flow cytometry-based assays.

### MSMD

Mendelian susceptibility to mycobacterial diseases (MSMD) is a group of approximately 30 different diseases associated with mutations in 15 genes, presenting inherited susceptibility to BCG and environmental atypical mycobacteriosis (285). The first diseases described in this group were defects in the expression of interferon-gamma alpha and beta chains (IFNGR1 and IFNGR2), followed by deficiencies in interleukin-12/23 beta 1 chain (IL12RB1) and STAT-1. Some of these diseases can be evaluated by flow cytometric expression of the molecules ex vivo or after stimulation.

#### Cell Surface and Cytoplasmic Protein Expression: IFNγ-R1, IFNγ-R2, IL12-RB1 and STAT-1

The first described diseases among MSMD, namely, IFNγ receptor and IL-12/23 receptor beta chain 1 deficiencies, can be easily evaluated by flow cytometry (286). IFNγ receptor alpha chain (IFNγ-R1 or CD119) can be evaluated by the expression of CD119 on monocytes, and T lymphocytes. Partial dominant negative IFNγ−R1 deficiency is usually characterized by overexpression of the receptor due to the lack of an intracellular domain region associated with impaired recycling of the molecule (287). Phosphorylated STAT-1 expression can be assessed by intracellular flow cytometry (288). Deficiency in the beta-1 chain of IL12/23 receptor (IL12RB1 or CD212) is the most common form of MSMD (289) and can be evaluated by flow cytometry after activation of T cells, somehow increasing the complexity of the evaluation and the possibility of standardization. The same approach is important to the evaluation of IL12RB2 protein expression, but this disease is very rare and has been described only recently (285). As for most of the extremely rare conditions described above, no protocols for test standardization and quality assessment programs are available to date (290).

### LAD

Leukocyte adhesion deficiency (LAD) syndromes are very rare autosomal recessive diseases characterized by leukocytosis associated or not with other clinical and laboratory features (291). There are three forms of LAD, namely, LAD1, 2 and 3, with different genetics and pathophysiology (292, 293). LAD1 is associated with mutations in *ITGB2*, which is the gene for the beta chain of beta-2 integrins, also known as CD18, and mediates cell-cell and cell-extracellular matrix adhesion (294, 295).Therefore, LAD1 patients present leukocytosis with neutrophilia associated with recurrent bacterial infections and impaired pus formation and wound healing (296). LAD2 is associated with the mutation of *SLC35C1*, which is a gene encoding a GDP-fucose transmembrane transporter (FucT1). It is characterized by leukocyte adhesion defects associated with severe mental and growth retardation. LAD2 is also known as a congenital disorder of glycosylation type IIc (297). LAD3 is caused by mutations in the *FERMT3* (or *KINDLIN3*) gene, presenting a leukocyte adhesion defect with delayed umbilical cord detaching, omphalitis, severe bacterial infections, and delayed wound healing and associated with bleeding tendency with normal platelet numbers (298).

#### Cell Surface Protein Expression: CD18, CD11a/CD11b/CD11c, CD15

Screening of leukocyte adhesion defects can be performed by simple flow cytometry techniques (299). CD18 is present in all lineages of nucleated hematopoietic cells, and its absence is typical of LAD1. LAD2 can be diagnosed by the presence of the Bombay phenotype due to the absence of the H antigen in red blood cells, therefore applying to blood types A, B, AB and O. Another characteristic is the absence of CD15s (sialyl-Lewis Ag) in flow cytometry. Finally, LAD3 can be screened by the absence of beta-1 and beta-2 integrins in flow cytometry of platelets and phagocytes. However, to our knowledge, none of these methodologies are standardized or have any quality assessment programs available.

## Conclusions

Standardization and quality assessment programs are pivotal for immunology diagnostic tests, especially those targeting “lab-dependent” identification of disorders routinely assessed by IEI-specialized clinical immunologists. Nonfunctional tests are generally a good alternative for relatively low-cost, quick and definitive diagnoses. Despite the rich literature describing anecdotal cases or series reports for each specific assay shown in this manuscript, unfortunately, most lack robust methodological and populational standardization. **Table 6** summarizes all nonfunctional immunoassays herein listed and stratifies them according to the presence or absence of standardization and quality assessment initiatives. The popularization and reliability of nonfunctional immunoassays should be enhanced by multicenter collaborative studies addressing methodological standardization and the establishment of reference ranges and quality assessment programs.

**Table 6:** Quality and standardization control stratification of inborn errors of immunity (IEI) assessment nonfunctional immunoassays.

IEI categories	Nonfunctional immunoassay	Method	Method standardization	Quality control program	Reference range (including early age groups)
Predominantly antibody deficiencies	IgG, IgM and IgA	Nephelometry or turbidimetry	Standardized	Established	Standardized
	IgD	ELISA	Standardized	Established	Nonstandardized
	IgE	ELISA or fluorimetry	Standardized	Established	Few initiatives
	IgG subclasses	Nephelometry or turbidimetry	Standardized	Established	Standardized
	Salivary IgA	ELISA	Few initiatives	Unestablished	Few initiatives
	Vaccine response against tetanus toxoid	ELISA	Standardized	Unestablished	Few initiatives
	Vaccine response against diphtheria toxoid	ELISA	Standardized	Unestablished	Few initiatives
	Vaccine response against measles	ELISA	Standardized	Unestablished	Standardized
	Vaccine response against mumps	ELISA	Standardized	Unestablished	Standardized
	*In vivo* vaccine response against *Streptococcus pneumoniae*	ELISA	Standardized	Established	Standardized
		Multiplex	Standardized	Unestablished	Nonstandardized
	Isohemagglutinins	Hemagglutination	Standardized	Unestablished	Few initiatives
	B cells (CD19^+^ or CD20^+^)	Flow cytometry	Standardized	Established	Standardized
	B cell immune phenotyping	Flow cytometry	Few initiatives	Unestablished	Few initiatives
	Intracellular BTK expression	Flow cytometry	Nonstandardized	Unestablished	Nonstandardized
	Defective cell surface CVID-related protein expression	Flow cytometry	Nonstandardized	Unestablished	Nonstandardized
Combined immunodeficiencies	TREC	qRT-PCR	Standardized	Unestablished	Few initiatives
	CD4^+^/CD8^+^ T cells	Flow cytometry	Standardized	Established	Standardized
	T cell immune phenotyping	Flow cytometry	Few initiatives	Unestablished	Few initiatives
	Th17 immunophenotyping	Flow cytometry	Nonstandardized	Unestablished	Few initiatives
	Intracellular Wiskott-Aldrich protein expression	Flow cytometry	Nonstandardized	Unestablished	Nonstandardized
	Defective cell surface or intracellular SCID-related protein expression	Flow cytometry	Nonstandardized	Unestablished	Nonstandardized
	ADA-1 erythrocyte activity	Fluorometry, spectrophotometry	Nonstandardized	Unestablished	Nonstandardized
	PNP erythrocyte activity	LCTMS, ECA	Nonstandardized	Unestablished	Nonstandardized
Diseases of immune dysregulation	Soluble CD25	ELISA, chemoluminescence	Standardized	Unestablished	Few initiatives
	Defective intracellular HLH-related protein expression (PRF1, SAP/SH2DIA, XIAP)	Flow cytometry	Nonstandardized	Unestablished	Nonstandardized
	Double negative TCRα/β circulating T cells (DNT)	Flow cytometry	Nonstandardized	Unestablished	Nonstandardized
	IL-10	ELISA, ALBIA	Standardized	Unestablished	Few initiatives
	IL-18	ELISA, ALBIA	Standardized	Unestablished	Few initiatives
	Soluble FASL	ELISA, ALBIA	Standardized	Unestablished	Few initiatives
	B12 vitamin	ELISA	Standardized	Established	Standardized
Defects in phagocytes, intrinsic and innate immunity	Cell surface protein expression: IFNγ-R1 and IFNγ-R2	Flow cytometry	Nonstandardized	Unestablished	Nonstandardized
	Cell surface protein expression: CD18, CD11a/CD11b/CD11c, CD15	Flow cytometry	Nonstandardized	Unestablished	Nonstandardized

The main methodologies platforms used for each test are also presented. Standardization and quality control stratification for each test are rated as: “standardized/established”, in the case of robust, clear data available; “few initiatives”, in the case of only reports or low-numbered uncontrolled case series available; or “nonstandardized/unestablished”, in the case of no trustworthy data available.

ADA-1, adenosine deaminase-1; ALBIA, addressable laser bead immunoassay; ECA, enzymatic colorimetric assay; ELISA, enzyme-linked immunosorbent assay; HLH, hemophagocytic lymphohystiocytosis; LCTMS, liquid chromatography-tandem mass spectrometry; PNP, purine nucleoside phosphorylase; PRF1, perforin 1; SAP/SH2DIA, SLAM-associated protein/SH2 domain–containing protein 1A; TREC, T cell receptor excision circles; XIAP, X-linked inhibitor of apoptosis.

## Author Contributions

SP, PP, DM-V, JO, LA, and MC-S contributed to conception and design of the study. SP organized the contents and wrote the first draft of the manuscript. All authors wrote sections of the manuscript. All authors contributed to manuscript revision, read, and approved the submitted version.

## Funding

Sao Paulo Research Agency (FAPESP) grant# 2014/50489-9 supported this study.

## Conflict of Interest

The authors declare that the research was conducted in the absence of any commercial or financial relationships that could be construed as a potential conflict of interest.

## Publisher’s Note

All claims expressed in this article are solely those of the authors and do not necessarily represent those of their affiliated organizations, or those of the publisher, the editors and the reviewers. Any product that may be evaluated in this article, or claim that may be made by its manufacturer, is not guaranteed or endorsed by the publisher.

## Glossary

**Table:**

ADA-1	Adenosine deaminase 1
ALBIA	addressable laser bead immunoassay
ALPS	autoimmune lymphoproliferative syndrome
ATM	ataxia-telangiectasia
BTK	Bruton’s tyrosine kinase
CAP	College of American Pathologists
CCR7	C-C chemokine receptor 7
CI	confidence intervals
CID	combined immunodeficiencies
CLIA	Clinical Laboratory Improvement Amendments
ChLIA	chemiluminescent immuno-assay
CVID	common variable immunodeficiency
DNT	double negative TCR alpha/beta circulating T cells
DOCK8	dedicator of cytokinesis 8
EIU/mL	enzyme international units/mL
ELISA	enzyme-linked immunosorbent assay
EPEC	enteropathogenic *E. coli*
FDA	Food and drug administration
GOF	gain-of-function
HIDS	hyper-IgD syndrome
HLH	hemophagocytic lymphohistiocytosis
IEI	inborn errors of immunity
IPEX	immunedysregulation polyendocrinopathy enteropathy X-linked
IUIS	International Union of Immunological Societies
LAD	leukocyte adhesion deficiency
LOF	loss-of-function
MKD	mevalonate kinase deficiency
MSMD	Mendelian susceptibility to mycobacterial diseases
NIAID-DAIDS	National Institute of Allergy and Infectious Diseases Division of AIDS
NK	natural killer
PAD	predominantly antibody deficiency
PERISCOPE	PERtussIS COrrelates of Protection Europe
PID	primary immunodeficiency diseases
PIRD	primary immune regulatory disorders
PNP	purine nucleoside phosphorylase
PnPS	pneumococcal polysaccharide
PPV-23	pneumococcal prevalent 23 serotypes
QAS	quality assessment
qRT-PCR	quantitative real-time polymerase chain reaction
RALD	RAS-associated autoimmune leukoproliferative disorder
SCID	severe combined immunodeficiency
SD	standard deviation
sIgA	secretory IgA
SIgAD	selective IgA deficiency
SPAD	specific polysaccharide antibody deficiency
TCR	T cell receptor
TREC	T cell receptor excision circles
Treg	regulatory T cells
TYK2	tyrosine kinase 2
WAS	Wiskott-Aldrich syndrome
WASP	Wiskott-Aldrich syndrome protein
WHO	World Health Organization
XLA	X-linked agammaglobulinemia
XLP	X-linked lymphoproliferative syndrome

## References

[1] Chan AY Torgerson TR. Primary Immune Regulatory Disorders: A Growing Universe of Immune Dysregulation. Curr Opin Allergy Clin Immunol (2020) 20(6):582–90. doi: 10.1097/ACI.0000000000000689 PMC7769114 32941318

[2] Notarangelo LD Bacchetta R Casanova JL Su HC. Human Inborn Errors of Immunity: An Expanding Universe. Sci Immunol (2020) 5(49):eabb1662. doi: 10.1126/sciimmunol.abb1662 32651211 PMC7647049

[3] Boyle JM Buckley RH. Population Prevalence of Diagnosed Primary Immunodeficiency Diseases in the United States. J Clin Immunol (2007) 27(5):497–502. doi: 10.1007/s10875-007-9103-1 17577648

[4] Bousfiha AA Jeddane L Ailal F Benhsaien I Mahlaoui N Casanova JL. Primary Immunodeficiency Diseases Worldwide: More Common Than Generally Thought. J Clin Immunol (2013) 33(1):1–7. doi: 10.1007/s10875-012-9751-7 22847546

[5] Tangye SG Al-Herz W Bousfiha A Chatila T Cunningham-Rundles C Etzioni A. Human Inborn Errors of Immunity: 2019 Update on the Classification From the International Union of Immunological Societies Expert Committee. J Clin Immunol (2020) 40(1):24–64. doi: 10.1007/s10875-019-00737-x 31953710 PMC7082301

[6] Bousfiha A Jeddane L Picard C Al-Herz W Ailal F Chatila T. Human Inborn Errors of Immunity: 2019 Update of the IUIS Phenotypical Classification. J Clin Immunol (2020) 40(1):66–81. doi: 10.1007/s10875-020-00758-x 32048120 PMC7082388

[7] Condino-Neto A Sorensen RU Gómez Raccio AC King A Espinosa-Rosales FJ Franco JL. Current State and Future Perspectives of the Latin American Society for Immunodeficiencies (LASID). Allergol Immunopathol (Madr) (2015) 43(5):493–7. doi: 10.1016/j.aller.2014.05.007 25294607

[8] Tangye SG Al-Herz W Bousfiha A Cunningham-Rundles C Franco JL Holland SM. The Ever-Increasing Array of Novel Inborn Errors of Immunity: An Interim Update by the IUIS Committee. J Clin Immunol (2021) 41(3):666–79. doi: 10.1007/s10875-021-00980-1 PMC7889474 33598806

[9] Oliveira JB Fleisher TA. Laboratory Evaluation of Primary Immunodeficiencies. J Allergy Clin Immunol (2010) 125(2 Suppl 2):S297–305. doi: 10.1016/j.jaci.2009.08.043 PMC3412511 20042230

[10] Rosenzweig SD KFT. Laboratory Evaluation of Primary Immunodeficiency Disorders. In: KE S, editor. Stiehm’s Immune Deficiencies, 2nd ed. USA: Elsevier Inc (2020). p. 115–31.

[11] Jennings L Van Deerlin VM Gulley ML. Committee CoAPMPR. Recommended Principles and Practices for Validating Clinical Molecular Pathology Tests. Arch Pathol Lab Med (2009) 133(5):743–55. doi: 10.5858/133.5.743 19415949

[12] Carneiro-Sampaio M Moraes-Vasconcelos D Kokron CM Jacob CM Toledo-Barros M Dorna MB. Primary Immunodeficiency Diseases in Different Age Groups: A Report on 1,008 Cases From a Single Brazilian Reference Center. J Clin Immunol (2013) 33(4):716–24. doi: 10.1007/s10875-013-9865-6 23354909

[13] Bonilla FA Bernstein IL Khan DA Ballas ZK Chinen J Frank MM. Practice Parameter for the Diagnosis and Management of Primary Immunodeficiency. Ann Allergy Asthma Immunol (2005) 94(5 Suppl 1):S1–63. doi: 10.1016/S1081-1206(10)61142-8 15945566

[14] Bonilla FA Geha RS. Primary Immunodeficiency Diseases. J Allergy Clin Immunol (2003) 111(2 Suppl):S571–81. doi: 10.1067/mai.2003.86 12592303

[15] Jolliff CR Cost KM Stivrins PC Grossman PP Nolte CR Franco SM. Reference Intervals for Serum IgG, IgA, IgM, C3, and C4 as Determined by Rate Nephelometry. Clin Chem (1982) 28(1):126–8. doi: 10.1093/clinchem/28.1.126 7055895

[16] Bonilla FA Khan DA Ballas ZK Chinen J Frank MM Hsu JT. Practice Parameter for the Diagnosis and Management of Primary Immunodeficiency. J Allergy Clin Immunol (2015) 136(5):1186–205.e1-78. doi: 10.1016/j.jaci.2015.04.049 26371839

[17] Palmeira P Quinello C Silveira-Lessa AL Zago CA Carneiro-Sampaio M. IgG Placental Transfer in Healthy and Pathological Pregnancies. Clin Dev Immunol (2012) 2012:985646. doi: 10.1155/2012/985646 22235228 PMC3251916

[18] Vilela MMDS. Human Inborn Errors of Immunity (HIEI): Predominantly Antibody Deficiencies (PADs): If You Suspect it, You can Detect it. J Pediatr (Rio J) (2021) 97 Suppl 1:S67–74. doi: 10.1016/j.jped.2020.10.010 PMC9432301 33245895

[19] Grumach AS Jacob CM Pastorino AC. [IgA Deficiency: Clinical and Laboratory Evaluation of 60 Patients From the “Instituto Da Criança”]. Rev Assoc Med Bras (1992) (1998) 44(4):277–82. doi: 10.1590/s0104-42301998000400005 9852646

[20] van der Burg M Dalm Virgil A Weemaes C. Isotype Defects. In: Sullivan K Stiehm E, editors. Stiehm’s Immune Deficiencies Inborn Errors of Immunity, 2 ed. Elsevier: Philadelphia (2020). p. 523–36.

[21] Herrod HG. Management of the Patient With IgG Subclass Deficiency and/or Selective Antibody Deficiency. Ann Allergy (1993) 70(1):3–8. 8424594

[22] Schauer U Stemberg F Rieger CH Borte M Schubert S Riedel F. IgG Subclass Concentrations in Certified Reference Material 470 and Reference Values for Children and Adults Determined With the Binding Site Reagents. Clin Chem (2003) 49(11):1924–9. doi: 10.1373/clinchem.2003.022350 14578325

[23] Wahn V von Bernuth H. IgG Subclass Deficiencies in Children: Facts and Fiction. Pediatr Allergy Immunol (2017) 28(6):521–4. doi: 10.1111/pai.12757 28686792

[24] Agarwal S Cunningham-Rundles C. Assessment and Clinical Interpretation of Reduced IgG Values. Ann Allergy Asthma Immunol (2007) 99(3):281–3. doi: 10.1016/S1081-1206(10)60665-5 PMC3099256 17910333

[25] Brandtzaeg P. Synthesis and Secretion of Human Salivary Immunoglobulins. In: Garrett J Ekstrom J Anderson L, editors. Glandular Mechanisms of Salivary Secretion Frontiers of Oral Biology. Basel: Karger (1998). p. 167.

[26] Nagao AT Pilagallo M Pereira AB Carneiro-Sampaio M Hanson LA. Quantification of Salivary, Urinary and Fecal Secretory IgA, as Well as in Saliva Titers and Avidities of IgA Antibodies in Children Living at Different Levels of Antigenic Exposure and Undernutrition. Adv Exp Med Biol (1995) 371A:507–11. doi: 10.1007/978-1-4615-1941-6_106 8525977

[27] Weemaes C Klasen I Göertz J Beldhuis-Valkis M Olafsson O Haraldsson A. Development of Immunoglobulin A in Infancy and Childhood. Scand J Immunol (2003) 58(6):642–8. doi: 10.1111/j.1365-3083.2003.01344.x 14636420

[28] Jafarzadeh A Sadeghi M Karam GA Vazirinejad R. Salivary IgA and IgE Levels in Healthy Subjects: Relation to Age and Gender. Braz Oral Res (2010) 24(1):21–7. doi: 10.1590/S1806-83242010000100004 20339709

[29] Mandel ID Khurana HS. The Relation of Human Salivary Gamma A Globulin and Albumin to Flow Rate. Arch Oral Biol (1969) 14(12):1433–5. doi: 10.1016/0003-9969(69)90261-1 4983131

[30] Grönblad EA. Concentration of Immunoglobulins in Human Whole Saliva: Effect of Physiological Stimulation. Acta Odontol Scand (1982) 40(2):87–95. doi: 10.3109/00016358209041120 6954831

[31] Wan AK Seow WK Purdie DM Bird PS Walsh LJ Tudehope DI. Immunoglobulins in Saliva of Preterm and Full-Term Infants. Oral Microbiol Immunol (2003) 18(2):72–8. doi: 10.1034/j.1399-302X.2003.00044.x 12654094

[32] Müller F Frøland SS Hvatum M Radl J Brandtzaeg P. Both IgA Subclasses are Reduced in Parotid Saliva From Patients With AIDS. Clin Exp Immunol (1991) 83(2):203–9. doi: 10.1111/j.1365-2249.1991.tb05615.x PMC1535252 1899629

[33] Phalipon A Corthésy B. Novel Functions of the Polymeric Ig Receptor: Well Beyond Transport of Immunoglobulins. Trends Immunol (2003) 24(2):55–8. doi: 10.1016/S1471-4906(02)00031-5 12547499

[34] Quinello C Quintilio W Carneiro-Sampaio M Palmeira P. Passive Acquisition of Protective Antibodies Reactive With Bordetella Pertussis in Newborns *via* Placental Transfer and Breast-Feeding. Scand J Immunol (2010) 72(1):66–73. doi: 10.1111/j.1365-3083.2010.02410.x 20591078

[35] Hanson LA Korotkova M. The Role of Breastfeeding in Prevention of Neonatal Infection. Semin Neonatol (2002) 7(4):275–81. doi: 10.1053/siny.2002.0124 12401297

[36] Lawrence RM Lawrence RA. Breast Milk and Infection. Clin Perinatol (2004) 31(3):501–28. doi: 10.1016/j.clp.2004.03.019 PMC7133241 15325535

[37] Palmeira P Carneiro-Sampaio M. Immunology of Breast Milk. Rev Assoc Med Bras (1992) (2016) 62(6):584–93. doi: 10.1590/1806-9282.62.06.584 27849237

[38] Barros MD Carneiro-Sompaio MM. Milk Composition of Low Birth Weight Infants’ Mothers. Acta Paediatr Scand (1984) 73(5):693–4. doi: 10.1111/j.1651-2227.1984.tb09997.x 6485789

[39] Palmeira P Costa-Carvalho BT Arslanian C Pontes GN Nagao AT Carneiro-Sampaio MM. Transfer of Antibodies Across the Placenta and in Breast Milk From Mothers on Intravenous Immunoglobulin. Pediatr Allergy Immunol (2009) 20(6):528–35. doi: 10.1111/j.1399-3038.2008.00828.x 19220771

[40] Levan-Petit I Cardonna J Garcia M Migeon J Corbi C Preud’homme JL. Sensitive ELISA for Human Immunoglobulin D Measurement in Neonate, Infant, and Adult Sera. Clin Chem (2000) 46(6 Pt 1):876–8. doi: 10.1093/clinchem/46.6.876 10839785

[41] Overed-Sayer CL Mosedale DE Goodall M Grainger DJ. Measurement of Human Serum IgD Levels. Curr Protoc Immunol (2009) 85:2.9B.1–7. doi: 10.1002/0471142735.im0209bs85 19347847

[42] van der Hilst JC Frenkel J. Hyperimmunoglobulin D Syndrome in Childhood. Curr Rheumatol Rep (2010) 12(2):101–7. doi: 10.1007/s11926-010-0086-1 20425018

[43] van der Meer JW Simon A. The Challenge of Autoinflammatory Syndromes: With an Emphasis on Hyper-IgD Syndrome. Rheumatol (Oxford) (2016) 55(suppl 2):ii23–ii9. doi: 10.1093/rheumatology/kew351 27856657

[44] Sacco C Perna S Vicari D Alfò M Bauer CP Hoffman U. Growth Curves of “Normal” Serum Total IgE Levels Throughout Childhood: A Quantile Analysis in a Birth Cohort. Pediatr Allergy Immunol (2017) 28(6):525–34. doi: 10.1111/pai.12738 28544337

[45] Barbee RA Halonen M Lebowitz M Burrows B. Distribution of IgE in a Community Population Sample: Correlations With Age, Sex, and Allergen Skin Test Reactivity. J Allergy Clin Immunol (1981) 68(2):106–11. doi: 10.1016/0091-6749(81)90167-6 7251998

[46] Barbee RA Halonen M Kaltenborn W Lebowitz M Burrows B. A Longitudinal Study of Serum IgE in a Community Cohort: Correlations With Age, Sex, Smoking, and Atopic Status. J Allergy Clin Immunol (1987) 79(6):919–27. doi: 10.1016/0091-6749(87)90241-7 3584747

[47] Platts-Mills TAE Schuyler AJ Erwin EA Commins SP Woodfolk JA. IgE in the Diagnosis and Treatment of Allergic Disease. J Allergy Clin Immunol (2016) 137(6):1662–70. doi: 10.1016/j.jaci.2016.04.010 PMC5406226 27264001

[48] Cooper PJ Alexander N Moncayo AL Benitez SM Chico ME Vaca MG. Environmental Determinants of Total IgE Among School Children Living in the Rural Tropics: Importance of Geohelminth Infections and Effect of Anthelmintic Treatment. BMC Immunol (2008) 9:33. doi: 10.1186/1471-2172-9-33 18588694 PMC2459155

[49] Levin ME Le Souëf PN Motala C. Total IgE in Urban Black South African Teenagers: The Influence of Atopy and Helminth Infection. Pediatr Allergy Immunol (2008) 19(5):449–54. doi: 10.1111/j.1399-3038.2007.00663.x 18221478

[50] Zhang Q Boisson B Béziat V Puel A Casanova JL. Human Hyper-IgE Syndrome: Singular or Plural? Mamm Genome (2018) 29(7-8):603–17. doi: 10.1007/s00335-018-9767-2 PMC6317873 30094507

[51] Bergerson JRE Freeman AF. An Update on Syndromes With a Hyper-IgE Phenotype. Immunol Allergy Clin North Am (2019) 39(1):49–61. doi: 10.1016/j.iac.2018.08.007 30466772

[52] Al-Shaikhly T Ochs HD. Hyper IgE Syndromes: Clinical and Molecular Characteristics. Immunol Cell Biol (2019) 97(4):368–79. doi: 10.1111/imcb.12209 30264496

[53] Williams KW Milner JD Freeman AF. Eosinophilia Associated With Disorders of Immune Deficiency or Immune Dysregulation. Immunol Allergy Clin North Am (2015) 35(3):523–44. doi: 10.1016/j.iac.2015.05.004 PMC4688016 26209898

[54] Zago CA Jacob CM de Albuquerque Diniz EM Lovisolo SM Zerbini MC Dorna M. Autoimmune Manifestations in SCID Due to IL7R Mutations: Omenn Syndrome and Cytopenias. Hum Immunol (2014) 75(7):662–6. doi: 10.1016/j.humimm.2014.04.006 24759676

[55] Ferastraoaru D Jordakieva G Jensen-Jarolim E. The Other Side of the Coin: IgE Deficiency, a Susceptibility Factor for Malignancy Occurrence. World Allergy Organ J (2021) 14(1):100505. doi: 10.1016/j.waojou.2020.100505 33664932 PMC7887422

[56] Ammann AJ Cain WA Ishizaka K Hong R Good RA. Immunoglobulin E Deficiency in Ataxia-Telangiectasia. N Engl J Med (1969) 281(9):469–72. doi: 10.1056/NEJM196908282810904 4183711

[57] Levy Y Nakum A Segal N Monselise Y Danon YL. The Association of Selective IgA Deficiency and IgE Hypogammaglobulinemia. Allergy (2005) 60(6):836–8. doi: 10.1111/j.1398-9995.2005.00799.x 15876317

[58] Lawrence MG Palacios-Kibler TV Workman LJ Schuyler AJ Steinke JW Payne SC. Low Serum IgE Is a Sensitive and Specific Marker for Common Variable Immunodeficiency (CVID). J Clin Immunol (2018) 38(3):225–33. doi: 10.1007/s10875-018-0476-0 PMC5934300 29453744

[59] Eijsvoogel NB Hollegien MI Bok LA Derksen-Lubsen G Dikken FPJ Leenders ACAP. Lower Percentage of Allergic Sensitization in Children With Down Syndrome. Pediatr Allergy Immunol (2017) 28(8):852–7. doi: 10.1111/pai.12796 28881053

[60] Bonilla F. Vaccination of Immune-Deficient Patients. In: Sullivan K Stiehm E, editors. Stiehm’s Immune Deficiencies Inborn Errors of Immunity, 2 ed. Philadelphia: Elsevier (2020). p. 1157–73.

[61] Junqueira AL Tavares VR Martins RM Frauzino KV da Costa e Silva AM Minamisava R. Safety and Immunogenicity of Hepatitis B Vaccine Administered Into Ventrogluteal vs. Anterolateral Thigh Sites in Infants: A Randomised Controlled Trial. Int J Nurs Stud (2010) 47(9):1074–9. doi: 10.1016/j.ijnurstu.2010.01.009 20189173

[62] Marsh RA Orange JS. Antibody Deficiency Testing for Primary Immunodeficiency: A Practical Review for the Clinician. Ann Allergy Asthma Immunol (2019) 123(5):444–53. doi: 10.1016/j.anai.2019.08.012 31446132

[63] Ambrosino DM Siber GR Chilmonczyk BA Jernberg JB Finberg RW. An Immunodeficiency Characterized by Impaired Antibody Responses to Polysaccharides. N Engl J Med (1987) 316(13):790–3. doi: 10.1056/NEJM198703263161306 3493431

[64] Organization WH. Training Manual for Enzyme Linked Immunosorbent Assay for the Quantitation of Streptococcus Pneumoniae Serotype Specific IgG (Pn PS ELISA). A Guide to Procedures for Qualification of Materials and Analysis of Assay Performance Geneva. Switzerland: World Health Organization (2004). Available at: http://www.vaccine.uab.edu/ELISA%20Protocol.pdf.

[65] Sorensen RU Leiva LE. Measurement of Pneumococcal Polysaccharide Antibodies. J Clin Immunol (2014) 34(2):127–8. doi: 10.1007/s10875-013-9977-z 24337649

[66] Balloch A Licciardi PV Tang ML. Serotype-Specific Anti-Pneumococcal IgG and Immune Competence: Critical Differences in Interpretation Criteria When Different Methods are Used. J Clin Immunol (2013) 33(2):335–41. doi: 10.1007/s10875-012-9806-9 23054341

[67] Sorensen RU. A Critical View of Specific Antibody Deficiencies. Front Immunol (2019) 10:986. doi: 10.3389/fimmu.2019.00986 31118939 PMC6506784

[68] Paradiso P. Essential Criteria for Evaluation of Pneumococcal Conjugate Vaccine Candidates. Vaccine (2009) 27:C15–8. doi: 10.1016/j.vaccine.2009.06.008 19683657

[69] Bonilla FA. Update: Vaccines in Primary Immunodeficiency. J Allergy Clin Immunol (2018) 141(2):474–81. doi: 10.1016/j.jaci.2017.12.980 29288077

[70] Whaley MJ Rose C Martinez J Laher G Sammons DL Smith JP. Interlaboratory Comparison of Three Multiplexed Bead-Based Immunoassays for Measuring Serum Antibodies to Pneumococcal Polysaccharides. Clin Vaccine Immunol (2010) 17(5):862–9. doi: 10.1128/CVI.00022-10 PMC2863388 20335434

[71] Zhang X Simmerman K Yen-Lieberman B Daly TM. Impact of Analytical Variability on Clinical Interpretation of Multiplex Pneumococcal Serology Assays. Clin Vaccine Immunol (2013) 20(7):957–61. doi: 10.1128/CVI.00223-13 PMC3697459 23677324

[72] Daly TM Pickering JW Zhang X Prince HE Hill HR. Multilaboratory Assessment of Threshold Versus Fold-Change Algorithms for Minimizing Analytical Variability in Multiplexed Pneumococcal IgG Measurements. Clin Vaccine Immunol (2014) 21(7):982–8. doi: 10.1128/CVI.00235-14 PMC4097436 24807051

[73] Lopez B Bahuaud M Fieschi C Mehlal S Jeljeli M Rogeau S. Value of the Overall Pneumococcal Polysaccharide Response in the Diagnosis of Primary Humoral Immunodeficiencies. Front Immunol (2017) 8:1862. doi: 10.3389/fimmu.2017.01862 29326723 PMC5742330

[74] Sorensen RU Edgar JDM. Overview of Antibody-Mediated Immunity to S. Pneumoniae: Pneumococcal Infections, Pneumococcal Immunity Assessment, and Recommendations for IG Product Evaluation. Transfusion (2018) 58:3106–13. doi: 10.1111/trf.15044 30536434

[75] Bonilla FA Barlan I Chapel H Costa-Carvalho BT Cunningham-Rundles C de la Morena MT. International Consensus Document (ICON): Common Variable Immunodeficiency Disorders. J Allergy Clin Immunol Pract (2016) 4(1):38–59. doi: 10.1016/j.jaip.2015.07.025 26563668 PMC4869529

[76] Branch DR. Anti-A and Anti-B: What are They and Where do They Come From? Transfusion (2015) 55:S74–9. doi: 10.1111/trf.13087 26174901

[77] Godzisz J. Synthesis of Natural Allohemagglutinins of the ABO System in Healthy Children Aged 3 Months to 3 Years. Rev Fr Transfus Immunohematol (1979) 22(4):399–412. doi: 10.1016/S0338-4535(79)80034-3 538394

[78] Schaballie H Vermeulen F Verbinnen B Frans G Vermeulen E Proesmans M. Value of Allohaemagglutinins in the Diagnosis of a Polysaccharide Antibody Deficiency. Clin Exp Immunol (2015) 180(2):271–9. doi: 10.1111/cei.12571 PMC4408162 25516411

[79] Blanco E Pérez-Andrés M Arriba-Méndez S Contreras-Sanfeliciano T Criado I Pelak O. Age-Associated Distribution of Normal B-Cell and Plasma Cell Subsets in Peripheral Blood. J Allergy Clin Immunol (2018) 141(6):2208–19.e16. doi: 10.1016/j.jaci.2018.02.017 29505809

[80] Wiener D Shah S Malone J Lowell N Lowitt S Rowlands DT. Multiparametric Analysis of Peripheral Blood in the Normal Pediatric Population by Flow Cytometry. J Clin Lab Anal (1990) 4(3):175–9. doi: 10.1002/jcla.1860040305 2352053

[81] Reichert T DeBruyère M Deneys V Tötterman T Lydyard P Yuksel F. Lymphocyte Subset Reference Ranges in Adult Caucasians. Clin Immunol Immunopathol (1991) 60(2):190–208. doi: 10.1016/0090-1229(91)90063-G 1712687

[82] Kotylo PK Fineberg NS Freeman KS Redmond NL Charland C. Reference Ranges for Lymphocyte Subsets in Pediatric Patients. Am J Clin Pathol (1993) 100(2):111–5. doi: 10.1093/ajcp/100.2.111 8356941

[83] Kontny U Barrachina C Habermehl P Mannhardt W Zepp F Schofer O. Distribution of Lymphocyte Surface Antigens in Healthy Neonates. Eur J Pediatr (1994) 153(4):257–9. doi: 10.1007/BF01954514 8194558

[84] Dhaliwal JS Balasubramaniam T Quek CK Gill HK Nasuruddin BA. Reference Ranges for Lymphocyte Subsets in a Defined Malaysian Population. Singapore Med J (1995) 36(3):288–91. 8553095

[85] Roman S Moldovan I Călugăru A Regalia T Sulică A. Lymphocyte Subset Reference Ranges in Romanian Adult Caucasians. Rom J Intern Med (1995) 33(1-2):27–36. 8535349

[86] Kam KM Leung WL Kwok MY Hung MY Lee SS Mak WP. Lymphocyte Subpopulation Reference Ranges for Monitoring Human Immunodeficiency Virus-Infected Chinese Adults. Clin Diagn Lab Immunol (1996) 3(3):326–30. doi: 10.1128/cdli.3.3.326-330.1996 PMC170341 8705678

[87] Robinson M O’Donohoe J Dadian G Wankowicz A Barltrop D Hobbs JR. An Analysis of the Normal Ranges of Lymphocyte Subpopulations in Children Aged 5-13 Years. Eur J Pediatr (1996) 155(7):535–9. doi: 10.1007/BF01957900 8831073

[88] Comans-Bitter WM de Groot R van den Beemd R Neijens HJ Hop WC Groeneveld K. Immunophenotyping of Blood Lymphocytes in Childhood. Reference Values for Lymphocyte Subpopulations. J Pediatr (1997) 130(3):388–93. doi: 10.1016/s0022-3476(97)70200-2 9063413

[89] Huppert FA Solomou W O’Connor S Morgan K Sussams P Brayne C. Aging and Lymphocyte Subpopulations: Whole-Blood Analysis of Immune Markers in a Large Population Sample of Healthy Elderly Individuals. Exp Gerontol (1998) 33(6):593–600. doi: 10.1016/S0531-5565(98)00033-3 9789736

[90] Shahabuddin S al Ayed IH el-Rad MO Qureshi MI. Lymphocyte Subset Reference Ranges in Healthy Saudi Arabian Children. Pediatr Allergy Immunol (1998) 9(1):44–8. doi: 10.1111/j.1399-3038.1998.tb00300.x 9560843

[91] Santagostino A Garbaccio G Pistorio A Bolis V Camisasca G Pagliaro P. An Italian National Multicenter Study for the Definition of Reference Ranges for Normal Values of Peripheral Blood Lymphocyte Subsets in Healthy Adults. Haematologica (1999) 84(6):499–504. 10366792

[92] Al Qouzi A Al Salamah A Al Rasheed R Al Musalam A Al Khairy K Kheir O. Immunophenotyping of Peripheral Blood Lymphocytes in Saudi Men. Clin Diagn Lab Immunol (2002) 9(2):279–81. doi: 10.1128/cdli.9.2.279-281.2002 PMC119953 11874863

[93] Kaaba SA Al Fadhli S Khamis A. Reference Values of Lymphocyte Subsets in the Normal Healthy Adult Kuwaiti Arab Population. Immunol Lett (2002) 81(3):199–203. doi: 10.1016/S0165-2478(01)00347-9 11947925

[94] Swaminathan S Hanna LE Raja A Sankaran K Kumar AN. Age-Related Changes in Blood Lymphocyte Subsets of South Indian Children. Natl Med J India (2003) 16(5):249–52. 14680279

[95] Shearer WT Rosenblatt HM Gelman RS Oyomopito R Plaeger S Stiehm ER. Lymphocyte Subsets in Healthy Children From Birth Through 18 Years of Age: The Pediatric AIDS Clinical Trials Group P1009 Study. J Allergy Clin Immunol (2003) 112(5):973–80. doi: 10.1016/j.jaci.2003.07.003 14610491

[96] Ikincioğullari A Kendirli T Doğu F Eğin Y Reisli I Cin S. Peripheral Blood Lymphocyte Subsets in Healthy Turkish Children. Turk J Pediatr (2004) 46(2):125–30. 15214740

[97] Timová S Leonardi GS Hrubá F Lochman I Lochmanová A Erdei E. Immune System Parameters in Children of Central and Eastern Europe: The CESAR Study. Cent Eur J Public Health (2004) 12(3):119–25. 15508409

[98] Chng WJ Tan GB Kuperan P. Establishment of Adult Peripheral Blood Lymphocyte Subset Reference Range for an Asian Population by Single-Platform Flow Cytometry: Influence of Age, Sex, and Race and Comparison With Other Published Studies. Clin Diagn Lab Immunol (2004) 11(1):168–73. doi: 10.1128/CDLI.11.1.168-173.2004 PMC321350 14715565

[99] Bisset LR Lung TL Kaelin M Ludwig E Dubs RW. Reference Values for Peripheral Blood Lymphocyte Phenotypes Applicable to the Healthy Adult Population in Switzerland. Eur J Haematol (2004) 72(3):203–12. doi: 10.1046/j.0902-4441.2003.00199.x 14962239

[100] Yaman A Cetiner S Kibar F Taşova Y Seydaoğlu G Dündar IH. Reference Ranges of Lymphocyte Subsets of Healthy Adults in Turkey. Med Princ Pract (2005) 14(3):189–93. doi: 10.1159/000084638 15863994

[101] Jentsch-Ullrich K Koenigsmann M Mohren M Franke A. Lymphocyte Subsets’ Reference Ranges in an Age- and Gender-Balanced Population of 100 Healthy Adults–a Monocentric German Study. Clin Immunol (2005) 116(2):192–7. doi: 10.1016/j.clim.2005.03.020 15993366

[102] Das Gupta A Ochani Z. Single Platform Enumeration of Lymphocyte Subsets in Healthy Indians Aged Between 18 and 49 Years. Cytometry B Clin Cytom (2006) 70(5):361–2. doi: 10.1002/cyto.b.20113 16906546

[103] Branch S Broome H Abayomi A. Characteristics of Lymphocyte Subsets in a Normal Afro-Caribbean Population and the Implications in HIV Management. Afr J Med Med Sci (2006) 35:109–12. 18050783

[104] Al-Jabri AA Al-Shukaili AK Al-Rashdi ZT Ganguly SS. Reference Ranges for Lymphocyte Subsets in Healthy Adult Male Omanis. Saudi Med J (2008) 29(3):409–12. 18327369

[105] Jiao Y Qiu Z Xie J Li D Li T. Reference Ranges and Age-Related Changes of Peripheral Blood Lymphocyte Subsets in Chinese Healthy Adults. Sci China C Life Sci (2009) 52(7):643–50. doi: 10.1007/s11427-009-0086-4 19641869

[106] Shokouhi Shoormasti R Azimdoost A Saghafi S Movahhedi M Haghi Ashtiani MT Pourpak Z. Normal Range Determination of Lymphocytes Subsets in Normal Adults in Iran. Iran J Allergy Asthma Immunol (2011) 10(4):295–8. 22184273

[107] Wong WS Lo AW Siu LP Leung JN Tu SP Tai SW. Reference Ranges for Lymphocyte Subsets Among Healthy Hong Kong Chinese Adults by Single-Platform Flow Cytometry. Clin Vaccine Immunol (2013) 20(4):602–6. doi: 10.1128/CVI.00476-12 PMC3623424 23408529

[108] Al-Mawali A Pinto AD Al Busaidi R Al-Zakwani I. Lymphocyte Subsets: Reference Ranges in an Age- and Gender-Balanced Population of Omani Healthy Adults. Cytometry A (2013) 83(8):739–44. doi: 10.1002/cyto.a.22322 23839863

[109] Kamallou A Haji Abdolbaghi M Mohraz M Rasolinejad M Karbasi E Ansaripour B. Reference Values of Lymphocyte Sub-Populations in Healthy Human Immunodeficiency Virus-Negative Iranian Adults. Iran J Immunol (2014) 11(4):221–32. 10.22034/iji.2014.16783 25549590

[110] Choi J Lee SJ Lee YA Maeng HG Lee JK Kang YW. Reference Values for Peripheral Blood Lymphocyte Subsets in a Healthy Korean Population. Immune Netw (2014) 14(6):289–95. doi: 10.4110/in.2014.14.6.289 PMC4275386 25550695

[111] Valiathan R Deeb K Diamante M Ashman M Sachdeva N Asthana D. Reference Ranges of Lymphocyte Subsets in Healthy Adults and Adolescents With Special Mention of T Cell Maturation Subsets in Adults of South Florida. Immunobiology (2014) 219(7):487–96. doi: 10.1016/j.imbio.2014.02.010 24661720

[112] Valdiglesias V Sánchez-Flores M Maseda A Marcos-Pérez D Millán-Calenti JC Pásaro E. Lymphocyte Subsets in a Population of Nonfrail Elderly Individuals. J Toxicol Environ Health A (2015) 78(13-14):790–804. doi: 10.1080/15287394.2015.1051170 26167746

[113] Al-Thani A Hamdi WS Al-Marwani A Alnaqdy A Sharafeldin H. Reference Ranges of Lymphocyte Subsets in Healthy Qatari Adults. biomark Med (2015) 9(5):443–52. doi: 10.2217/bmm.14.83 25275858

[114] Jia L Li J Zhang Y Shi Y Yuan E Liu J. Age- and Sex-Related Reference Intervals of Lymphocyte Subsets in Healthy Ethnic Han Chinese Children. Cytometry A (2015) 87(12):1116–26. doi: 10.1002/cyto.a.22716 26155000

[115] Shahal-Zimra Y Rotem Z Chezar J Shochat T Ross L Pickholtz I. Lymphocyte Subset Reference Ranges in Healthy Israeli Adults. Isr Med Assoc J (2016) 18(12):739–43. 28457077

[116] Qin L Jing X Qiu Z Cao W Jiao Y Routy JP. Aging of Immune System: Immune Signature From Peripheral Blood Lymphocyte Subsets in 1068 Healthy Adults. Aging (Albany NY) (2016) 8(5):848–59. doi: 10.18632/aging.100894 PMC4931839 26886066

[117] Azarsiz E Karaca NE Aksu G Kutukculer N. Reference Values for B-Cell Surface Markers and Co-Receptors Associated With Primary Immune Deficiencies in Healthy Turkish Children. Int J Immunopathol Pharmacol (2017) 30(2):194–200. doi: 10.1177/0394632017707609 28449602 PMC5806800

[118] Kokuina E Breff-Fonseca MC Villegas-Valverde CA Mora-Díaz I. Normal Values of T, B and NK Lymphocyte Subpopulations in Peripheral Blood of Healthy Cuban Adults. MEDICC Rev (2019) 21(2-3):16–21. doi: 10.37757/MR2019.V21.N2-3.5 31373580

[119] El Allam A El Fakihi S Tahoune H Sahmoudi K Bousserhane H Bakri Y. Age-Stratified Pediatric Reference Values of Lymphocytes in the Moroccan Population. Hum Antibodies (2020) 29(1):85–94. doi: 10.3233/HAB-200432 33252069

[120] Lerkvaleekul B Apiwattanakul N Klinmalai C Hongeng S Vilaiyuk S. Age-Related Changes in Lymphocyte Subpopulations in Healthy Thai Children. J Clin Lab Anal (2020) 34(5):e23156. doi: 10.1002/jcla.23156 31855295 PMC7246386

[121] Homburger HA McCarthy R Deodhar S. Assessment of Interlaboratory Variability in Analytical Cytology. Results of the College of American Pathologists Flow Cytometry Study. Arch Pathol Lab Med (1989) 113(6):667–72. 2658910

[122] Paxton H Kidd P Landay A Giorgi J Flomenberg N Walker E. Results of the Flow Cytometry ACTG Quality Control Program: Analysis and Findings. Clin Immunol Immunopathol (1989) 52(1):68–84. doi: 10.1016/0090-1229(89)90194-3 2785890

[123] Rickman WJ Waxdal MJ Monical C Damato JD Burke DS. Department of Army Lymphocyte Immunophenotyping Quality Assurance Program. Clin Immunol Immunopathol (1989) 52(1):85–95. doi: 10.1016/0090-1229(89)90195-5 2721036

[124] Brando B Sommaruga E. Nationwide Quality Control Trial on Lymphocyte Immunophenotyping and Flow Cytometer Performance in Italy. Cytometry (1993) 14(3):294–306. doi: 10.1002/cyto.990140310 8472606

[125] Goguel AF Crainic K Ducailar A Ouin M. Interlaboratory Quality Assessment of Lymphocyte Phenotyping. Etalonorme 1990-1992 Surveys. Biol Cell (1993) 78(1-2):79–84. doi: 10.1016/0248-4900(93)90118-x 8220229

[126] Van Blerk M Bernier M Bossuyt X Chatelain B D’Hautcourt JL Demanet C. National External Quality Assessment Scheme for Lymphocyte Immunophenotyping in Belgium. Clin Chem Lab Med (2003) 41(3):323–30. doi: 10.1515/CCLM.2003.052 12705342

[127] Levering WH van Wieringen WN Kraan J van Beers WA Sintnicolaas K van Rhenen DJ. Flow Cytometric Lymphocyte Subset Enumeration: 10 Years of External Quality Assessment in the Benelux Countries. Cytometry B Clin Cytom (2008) 74(2):79–90. doi: 10.1002/cyto.b.20370 17849485

[128] Sanz I Wei C Lee FE Anolik J. Phenotypic and Functional Heterogeneity of Human Memory B Cells. Semin Immunol (2008) 20(1):67–82. doi: 10.1016/j.smim.2007.12.006 18258454 PMC2440717

[129] Wehr C Eibel H Masilamani M Illges H Schlesier M Peter HH. A New CD21low B Cell Population in the Peripheral Blood of Patients With SLE. Clin Immunol (2004) 113(2):161–71. doi: 10.1016/j.clim.2004.05.010 15451473

[130] Isnardi I Ng YS Menard L Meyers G Saadoun D Srdanovic I. Complement Receptor 2/CD21- Human Naive B Cells Contain Mostly Autoreactive Unresponsive Clones. Blood (2010) 115(24):5026–36. doi: 10.1182/blood-2009-09-243071 PMC3373152 20231422

[131] Piątosa B Wolska-Kuśnierz B Pac M Siewiera K Gałkowska E Bernatowska E. B Cell Subsets in Healthy Children: Reference Values for Evaluation of B Cell Maturation Process in Peripheral Blood. Cytometry B Clin Cytom (2010) 78(6):372–81. doi: 10.1002/cyto.b.20536 20533385

[132] Morbach H Eichhorn EM Liese JG Girschick HJ. Reference Values for B Cell Subpopulations From Infancy to Adulthood. Clin Exp Immunol (2010) 162(2):271–9. doi: 10.1111/j.1365-2249.2010.04206.x PMC2996594 20854328

[133] Kverneland AH Streitz M Geissler E Hutchinson J Vogt K Boës D. Age and Gender Leucocytes Variances and References Values Generated Using the Standardized ONE-Study Protocol. Cytometry A (2016) 89(6):543–64. doi: 10.1002/cyto.a.22855 27144459

[134] Garcia-Prat M Álvarez-Sierra D Aguiló-Cucurull A Salgado-Perandrés S Briongos-Sebastian S Franco-Jarava C. Extended Immunophenotyping Reference Values in a Healthy Pediatric Population. Cytometry B Clin Cytom (2019) 96(3):223–33. doi: 10.1002/cyto.b.21728 30334372

[135] Kanegane H Futatani T Wang Y Nomura K Shinozaki K Matsukura H. Clinical and Mutational Characteristics of X-Linked Agammaglobulinemia and its Carrier Identified by Flow Cytometric Assessment Combined With Genetic Analysis. J Allergy Clin Immunol (2001) 108(6):1012–20. doi: 10.1067/mai.2001.120133 11742281

[136] Futatani T Miyawaki T Tsukada S Hashimoto S Kunikata T Arai S. Deficient Expression of Bruton’s Tyrosine Kinase in Monocytes From X-Linked Agammaglobulinemia as Evaluated by a Flow Cytometric Analysis and its Clinical Application to Carrier Detection. Blood (1998) 91(2):595–602. doi: 10.1182/blood.V91.2.595 9427714

[137] Martinez-Gallo M Radigan L Almejún MB Martínez-Pomar N Matamoros N Cunningham-Rundles C. TACI Mutations and Impaired B-Cell Function in Subjects With CVID and Healthy Heterozygotes. J Allergy Clin Immunol (2013) 131(2):468–76. doi: 10.1016/j.jaci.2012.10.029 PMC3646641 23237420

[138] Warnatz K Schlesier M. Flowcytometric Phenotyping of Common Variable Immunodeficiency. Cytometry B Clin Cytom (2008) 74(5):261–71. doi: 10.1002/cyto.b.20432 18561200

[139] Bunk R Dittrich AM Schulze I Horn J Schmolke K Volk HD. Rapid Whole Blood Flow Cytometric Test to Detect ICOS Deficiency in Patients With Common Variable Immunodeficiency. Int Arch Allergy Immunol (2006) 140(4):342–4. doi: 10.1159/000093770 16757923

[140] Rosain J Miot C Lambert N Rousselet MC Pellier I Picard C. CD21 Deficiency in 2 Siblings With Recurrent Respiratory Infections and Hypogammaglobulinemia. J Allergy Clin Immunol Pract (2017) 5(6):1765–7.e3. doi: 10.1016/j.jaip.2017.04.011 28499783

[141] Wentink MW Lambeck AJ van Zelm MC Simons E van Dongen JJ IJspeert H. CD21 and CD19 Deficiency: Two Defects in the Same Complex Leading to Different Disease Modalities. Clin Immunol (2015) 161(2):120–7. doi: 10.1016/j.clim.2015.08.010 26325596

[142] Roussel L Landekic M Golizeh M Gavino C Zhong MC Chen J. Loss of Human ICOSL Results in Combined Immunodeficiency. J Exp Med (2018) 215(12):3151–64. doi: 10.1084/jem.20180668 PMC6279397 30498080

[143] Griffith LM Cowan MJ Notarangelo LD Puck JM Buckley RH Candotti F. Improving Cellular Therapy for Primary Immune Deficiency Diseases: Recognition, Diagnosis, and Management. J Allergy Clin Immunol (2009) 124(6):1152–60.e12. doi: 10.1016/j.jaci.2009.10.022 20004776 PMC2831471

[144] Griffith LM Cowan MJ Notarangelo LD Kohn DB Puck JM Shearer WT. Primary Immune Deficiency Treatment Consortium (PIDTC) Update. J Allergy Clin Immunol (2016) 138(2):375–85. doi: 10.1016/j.jaci.2016.01.051 PMC4986691 27262745

[145] Chan K Puck JM. Development of Population-Based Newborn Screening for Severe Combined Immunodeficiency. J Allergy Clin Immunol (2005) 115(2):391–8. doi: 10.1016/j.jaci.2004.10.012 15696101

[146] Puck JM. Laboratory Technology for Population-Based Screening for Severe Combined Immunodeficiency in Neonates: The Winner is T-Cell Receptor Excision Circles. J Allergy Clin Immunol (2012) 129(3):607–16. doi: 10.1016/j.jaci.2012.01.032 PMC3294074 22285280

[147] Kwan A Abraham RS Currier R Brower A Andruszewski K Abbott JK. Newborn Screening for Severe Combined Immunodeficiency in 11 Screening Programs in the United States. JAMA (2014) 312(7):729–38. doi: 10.1001/jama.2014.9132 PMC4492158 25138334

[148] Horvath D Kayser C Silva CA Terreri MT Hilário MO Andrade LE. Decreased Recent Thymus Emigrant Number in Rheumatoid Factor-Negative Polyarticular Juvenile Idiopathic Arthritis. Clin Exp Rheumatol (2010) 28(3):348–53. 20460033

[149] Levy-Mendelovich S Lev A Aviner S Rosenberg N Kaplinsky C Sharon N. Quantification of Specific T and B Cells Immunological Markers in Children With Chronic and Transient ITP. Pediatr Blood Cancer (2017) 64(12):e27058. doi: 10.1002/pbc.26646 28544224

[150] Douek DC Vescio RA Betts MR Brenchley JM Hill BJ Zhang L. Assessment of Thymic Output in Adults After Haematopoietic Stem-Cell Transplantation and Prediction of T-Cell Reconstitution. Lancet (2000) 5(9218):1875–81. doi: 10.1016/S0140-6736(00)02293-5 10866444

[151] Hazenberg MD Otto SA Cohen Stuart JW Verschuren MC Borleffs JC Boucher CA. Increased Cell Division But Not Thymic Dysfunction Rapidly Affects the T-Cell Receptor Excision Circle Content of the Naive T Cell Population in HIV-1 Infection. Nat Med (2000) 6(9):1036–42. doi: 10.1038/79549 10973325

[152] Douek DC McFarland RD Keiser PH Gage EA Massey JM Haynes BF. Changes in Thymic Function With Age and During the Treatment of HIV Infection. Nature (1998) 396(6712):690–5. doi: 10.1038/25374 9872319

[153] Hazenberg MD Verschuren MC Hamann D Miedema F van Dongen JJ. T Cell Receptor Excision Circles as Markers for Recent Thymic Emigrants: Basic Aspects, Technical Approach, and Guidelines for Interpretation. J Mol Med (Berl) (2001) 79(11):631–40. doi: 10.1007/s001090100271 11715066

[154] Levy A Rangel-Santos A Torres LC Silveira-Abreu G Agena F Carneiro-Sampaio M. T Cell Receptor Excision Circles as a Tool for Evaluating Thymic Function in Young Children. Braz J Med Biol Res (2019) 52(7):e8292. doi: 10.1590/1414-431x20198292 31241713 PMC6596370

[155] Lang PO Govind S Dramé M Aspinall R. Measuring the TREC Ratio in Dried Blood Spot Samples: Intra- and Inter-Filter Paper Cards Reproducibility. J Immunol Methods (2013) 389(1-2):1–8. doi: 10.1016/j.jim.2012.12.003 23313293

[156] Serana F Chiarini M Zanotti C Sottini A Bertoli D Bosio A. Use of V(D)J Recombination Excision Circles to Identify T- and B-Cell Defects and to Monitor the Treatment in Primary and Acquired Immunodeficiencies. J Transl Med (2013) 11:119. doi: 10.1186/1479-5876-11-119 23656963 PMC3666889

[157] Borte S von Döbeln U Fasth A Wang N Janzi M Winiarski J. Neonatal Screening for Severe Primary Immunodeficiency Diseases Using High-Throughput Triplex Real-Time PCR. Blood (2012) 119(11):2552–5. doi: 10.1182/blood-2011-08-371021 22130802

[158] Baker MW Grossman WJ Laessig RH Hoffman GL Brokopp CD Kurtycz DF. Development of a Routine Newborn Screening Protocol for Severe Combined Immunodeficiency. J Allergy Clin Immunol (2009) 124(3):522–7. doi: 10.1016/j.jaci.2009.04.007 19482345

[159] Barbaro M Ohlsson A Borte S Jonsson S Zetterström RH King J. Newborn Screening for Severe Primary Immunodeficiency Diseases in Sweden-A 2-Year Pilot TREC and KREC Screening Study. J Clin Immunol (2017) 37(1):51–60. doi: 10.1007/s10875-016-0347-5 27873105 PMC5226987

[160] Dorsey MJ Puck JM. Newborn Screening for Severe Combined Immunodeficiency in the United States: Lessons Learned. Immunol Allergy Clin North Am (2019) 39(1):1–11. doi: 10.1016/j.iac.2018.08.002 30466767

[161] Denny T Yogev R Gelman R Skuza C Oleske J Chadwick E. Lymphocyte Subsets in Healthy Children During the First 5 Years of Life. JAMA (1992) 267(11):1484–8. doi: 10.1001/jama.1992.03480110060034 1347086

[162] Howard RR Fasano CS Frey L Miller CH. Reference Intervals of CD3, CD4, CD8, CD4/CD8, and Absolute CD4 Values in Asian and Non-Asian Populations. Cytometry (1996) 26(3):231–2. doi: 10.1002/(SICI)1097-0320(19960915)26:3<231::AID-CYTO9>3.0.CO;2-H 8889397

[163] Lisse IM Aaby P Whittle H Jensen H Engelmann M Christensen LB. T-Lymphocyte Subsets in West African Children: Impact of Age, Sex, and Season. J Pediatr (1997) 130(1):77–85. doi: 10.1016/S0022-3476(97)70313-5 9003854

[164] Tsegaye A Messele T Tilahun T Hailu E Sahlu T Doorly R. Immunohematological Reference Ranges for Adult Ethiopians. Clin Diagn Lab Immunol (1999) 6(3):410–4. doi: 10.1128/CDLI.6.3.410-414.1999 PMC103732 10225845

[165] Uppal SS Verma S Dhot PS. Normal Values of CD4 and CD8 Lymphocyte Subsets in Healthy Indian Adults and the Effects of Sex, Age, Ethnicity, and Smoking. Cytometry B Clin Cytom (2003) 52(1):32–6. doi: 10.1002/cyto.b.10011 12599179

[166] Lugada ES Mermin J Kaharuza F Ulvestad E Were W Langeland N. Population-Based Hematologic and Immunologic Reference Values for a Healthy Ugandan Population. Clin Diagn Lab Immunol (2004) 11(1):29–34. doi: 10.1128/CDLI.11.1.29-34.2004 14715541 PMC321349

[167] Bussmann H Wester CW Masupu KV Peter T Gaolekwe SM Kim S. Low CD4+ T-Lymphocyte Values in Human Immunodeficiency Virus-Negative Adults in Botswana. Clin Diagn Lab Immunol (2004) 11(5):930–5. doi: 10.1128/CDLI.11.5.930-935.2004 PMC515279 15358655

[168] Jiang W Kang L Lu HZ Pan X Lin Q Pan Q. Normal Values for CD4 and CD8 Lymphocyte Subsets in Healthy Chinese Adults From Shanghai. Clin Diagn Lab Immunol (2004) 11(4):811–3. doi: 10.1128/CDLI.11.4.811-813.2004 PMC440627 15242966

[169] Amatya R Vajpayee M Kaushik S Kanswal S Pandey RM Seth P. Lymphocyte Immunophenotype Reference Ranges in Healthy Indian Adults: Implications for Management of HIV/AIDS in India. Clin Immunol (2004) 112(3):290–5. doi: 10.1016/j.clim.2004.04.008 15308123

[170] Gomo E Vennervald BJ Ndhlovu P Kaestel P Nyazema N Friis H. Predictors and Reference Values of CD4 and CD8 T Lymphocyte Counts in Pregnancy: A Cross Sectional Study Among HIV Negative Women in Zimbabwe. Cent Afr J Med (2004) 50(1-2):10–9. 15490719

[171] Aina O Dadik J Charurat M Amangaman P Gurumdi S Mang E. Reference Values of CD4 T Lymphocytes in Human Immunodeficiency Virus-Negative Adult Nigerians. Clin Diagn Lab Immunol (2005) 12(4):525–30. doi: 10.1128/CDLI.12.4.525-530.2005 PMC1074389 15817761

[172] Ampofo W Torpey K Mukadi YD Koram K Nolan K Amenyah R. Normal CD4+ T Lymphocyte Levels in HIV Seronegative Individuals in the Manya/Yilo Krobo Communities in the Eastern Region of Ghana. Viral Immunol (2006) 19(2):260–6. doi: 10.1089/vim.2006.19.260 16817768

[173] Klose N Coulibaly B Tebit DM Nauwelaers F Spengler HP Kynast-Wolf G. Immunohematological Reference Values for Healthy Adults in Burkina Faso. Clin Vaccine Immunol (2007) 14(6):782–4. doi: 10.1128/CVI.00044-07 PMC1951086 17442846

[174] Das BR Bhanushali AA Khadapkar R Jeswani KD Bhavsar M Dasgupta A. Reference Ranges for Lymphocyte Subsets in Adults From Western India: Influence of Sex, Age and Method of Enumeration. Indian J Med Sci (2008) 62(10):397–406. doi: 10.4103/0019-5359.42725 19008613

[175] Ngowi BJ Mfinanga SG Bruun JN Morkve O. Immunohaematological Reference Values in Human Immunodeficiency Virus-Negative Adolescent and Adults in Rural Northern Tanzania. BMC Infect Dis (2009) 9:1. doi: 10.1186/1471-2334-9-1 19144106 PMC2630915

[176] Murugavel KG Balakrishnan P Mohanakrishnan J Solomon SS Shankar EM Muthu Sundaram SP. Establishment of T-Lymphocyte Subset Reference Intervals in a Healthy Adult Population in Chennai, India. Indian J Med Res (2009) 129(1):59–63. 19287058

[177] Chama CM Morrupa JY Abja UA Kayode A. Normal CD4 T-Lymphocyte Baseline in Healthy HIV-Negative Pregnant Women. J Obstet Gynaecol (2009) 29(8):702–4. doi: 10.3109/01443610903182920 19821661

[178] Oladepo DK Idigbe EO Audu RA Inyang US Imade GE Philip AO. Establishment of Reference Values of CD4 and CD8 Lymphocyte Subsets in Healthy Nigerian Adults. Clin Vaccine Immunol (2009) 16(9):1374–7. doi: 10.1128/CVI.00378-08 PMC2745013 19641097

[179] Lawrie D Coetzee LM Becker P Mahlangu J Stevens W Glencross DK. Local Reference Ranges for Full Blood Count and CD4 Lymphocyte Count Testing. S Afr Med J (2009) 99(4):243–8. 19588777

[180] Buchanan AM Muro FJ Gratz J Crump JA Musyoka AM Sichangi MW. Establishment of Haematological and Immunological Reference Values for Healthy Tanzanian Children in Kilimanjaro Region. Trop Med Int Health (2010) 15(9):1011–21. doi: 10.1111/j.1365-3156.2010.02585.x PMC3024440 20636301

[181] Sagnia B Ateba Ndongo F Ndiang Moyo Tetang S Ndongo Torimiro J Cairo C Domkam I. Reference Values of Lymphocyte Subsets in Healthy, HIV-Negative Children in Cameroon. Clin Vaccine Immunol (2011) 18(5):790–5. doi: 10.1128/CVI.00483-10 PMC3122514 21411603

[182] Thakar MR Abraham PR Arora S Balakrishnan P Bandyopadhyay B Joshi AA. Establishment of Reference CD4+ T Cell Values for Adult Indian Population. AIDS Res Ther (2011) 8:35. doi: 10.1186/1742-6405-8-35 21967708 PMC3198876

[183] Pennap GR Adoga MP Forbi JC Ojobo F Agwale SM. CD4+ T Lymphocyte Reference Values of Immunocompetent Subjects in an African University. Trop Doct (2011) 41(4):218–21. doi: 10.1258/td.2011.110219 21914674

[184] Adoga MP Pennap GR John PA Shawulu PT Kaba SV Forbi JC. CD4- and CD3-T Lymphocyte Reference Values of Immunocompetent Urban and Rural Subjects in an African Nation. Scand J Immunol (2012) 76(1):33–8. doi: 10.1111/j.1365-3083.2012.02700.x 22686509

[185] Shakya G Dumre SP Malla S Sharma M Kc KP Chhetri DB. Values of Lymphocyte Subsets in Nepalese Healthy Adult Population. JNMA J Nepal Med Assoc (2012) 52(185):6–13. doi: 10.31729/jnma.45 23279766

[186] García-Dabrio MC Pujol-Moix N Martinez-Perez A Fontcuberta J Souto JC Soria JM. Influence of Age, Gender and Lifestyle in Lymphocyte Subsets: Report From the Spanish Gait-2 Study. Acta Haematol (2012) 127(4):244–9. doi: 10.1159/000337051 22538526

[187] Touil N Hadef R Lemnouer A Zrara A Sbai AI Belfquih B. Range-Reference Determination of Lymphocyte Subsets in Moroccan Blood Donors. Afr Health Sci (2012) 12(3):334–8. doi: 10.4314/ahs.v12i3.14 PMC3557697 23382749

[188] Moreno-Galván M Palafox A. CD4+ CD8+ T Cell Reference Values in the Mexico City Population. Clin Vaccine Immunol (2013) 20(2):306–8. doi: 10.1128/CVI.00523-12 PMC3571261 23239806

[189] Torres AJ Angelo AL Silva MO Bastos M Souza DF Inocêncio LA. Establishing the Reference Range for T Lymphocytes Subpopulations in Adults and Children From Brazil. Rev Inst Med Trop Sao Paulo (2013) 55(5):323–8. doi: 10.1590/S0036-46652013000500005 PMC4105069 24037286

[190] Atanasova V Mihaylova A Lukanov T Atanasova M Nikolov A Naumova E. Evaluation of Lymphocyte Subpopulations in Cord Blood of Bulgarian Newborns. Clin Lab (2014) 60(11):1887–93. doi: 10.7754/Clin.Lab.2014.140107 25648031

[191] Tembe N Joaquim O Alfai E Sitoe N Viegas E Macovela E. Reference Values for Clinical Laboratory Parameters in Young Adults in Maputo, Mozambique. PloS One (2014) 9(5):e97391. doi: 10.1371/journal.pone.0097391 24827458 PMC4020854

[192] Prasetyo AA Zaini KU. Establishing Mean CD4+ T Cell Values Among Healthy Javanese Adults in Indonesia. Southeast Asian J Trop Med Public Health (2015) 46(4):662–8. 26867386

[193] Zhang K Wang F Zhang M Cao X Yang S Jia S. Reference Ranges of Lymphocyte Subsets Balanced for Age and Gender From a Population of Healthy Adults in Chongqing District of China. Cytometry B Clin Cytom (2016) 90(6):538–42. doi: 10.1002/cyto.b.21323 26352589

[194] Afolabi JK Fadeyi A Desalu OO Durotoye IA Fawibe AE Adeboye MAN. Normal CD4 Count Range Among Healthy Nigerian Population in Ilorin. J Int Assoc Provid AIDS Care (2017) 16(4):359–65. doi: 10.1177/2325957414530472 24842948

[195] Mulu W Abera B Mekonnen Z Adem Y Yimer M Zenebe Y. Haematological and CD4+ T Cells Reference Ranges in Healthy Adult Populations in Gojjam Zones in Amhara Region, Ethiopia. PloS One (2017) 12(7):e0181268. doi: 10.1371/journal.pone.0181268 28723945 PMC5516999

[196] Yeshanew AG GebreSilasie YM Mengesha HT. Establishment of Immunohematological Reference Values Among HIV Sero-Negative Pregnant Women at St. Paul’s Hospital Millennium Medical College (SPHMMC), Addis Ababa, Ethiopia. Ethiop J Health Sci (2017) 27(6):641–50. doi: 10.4314/ejhs.v27i6.9 PMC5811943 29487473

[197] Genetu M Damtie D Workineh M Mathewos Tebeje B Enawgaw B Deressa T. Immunological and Hematological Reference Intervals Among HIV-Seronegative Pregnant Women in Northwest Ethiopia. Int J Womens Health (2017) 9:145–50. doi: 10.2147/IJWH.S126916 PMC5344411 28424562

[198] Enawgaw B Birhan W Abebe M Terefe B Baynes HW Deressa T. Haematological and Immunological Reference Intervals for Adult Population in the State of Amhara, Ethiopia. Trop Med Int Health (2018) 23(7):765–73. doi: 10.1111/tmi.13071 29752840

[199] Karn S Bhattarai M Rauniyar R Adhikari A Karna P Upadhyay BP. Determination of CD4+ T- Lymphocytes in Healthy Children of Kathmandu. J Nepal Health Res Counc (2018) 16(3):325–9. doi: 10.33314/jnhrc.v16i3.1068 30455494

[200] Louati N Rekik T Menif H Gargouri J. Blood Lymphocyte T Subsets Reference Values in Blood Donors by Flow Cytometry. Tunis Med (2019) 97(2):327–34. 31539091

[201] Mishra SK Shrestha L Pandit R Khadka S Shrestha B Dhital S. Establishment of Reference Range of CD4 T-Lymphocyte in Healthy Nepalese Adults. BMC Res Notes (2020) 13(1):316. doi: 10.1186/s13104-020-05156-5 32616011 PMC7330941

[202] Niu HQ Zhao XC Li W Xie JF Liu XQ Luo J. Characteristics and Reference Ranges of CD4. BMC Immunol (2020) 21(1):44. doi: 10.1186/s12865-020-00374-9 32746780 PMC7397677

[203] Scheffer-Mendoza S Espinosa-Padilla SE López-Herrera G Mujica-Guzmán F López-Padilla MG Berrón-Ruiz L. Reference Values of Leukocyte and Lymphocytes Populations in Umbilical Cord and Capillary Blood in Healthy Mexican Newborns. Allergol Immunopathol (Madr) (2020) 48(3):295–305. doi: 10.1016/j.aller.2019.12.009 32312563

[204] Denny TN Gelman R Bergeron M Landay A Lam L Louzao R. A North American Multilaboratory Study of CD4 Counts Using Flow Cytometric Panleukogating (PLG): A NIAID-DAIDS Immunology Quality Assessment Program Study. Cytometry B Clin Cytom (2008) 74:S52–64. doi: 10.1002/cyto.b.20417 18351622

[205] Bainbridge J Wilkening CL Rountree W Louzao R Wong J Perza N. The Immunology Quality Assessment Proficiency Testing Program for CD3⁺4⁺ and CD3⁺8⁺ Lymphocyte Subsets: A Ten Year Review *via* Longitudinal Mixed Effects Modeling. J Immunol Methods (2014) 409:82–90. doi: 10.1016/j.jim.2014.05.017 24911327 PMC4148146

[206] Barnett D Whitby L Wong J Louzao R Reilly JT Denny TN. VERITAS?: A Time for VERIQAS™ and a New Approach to Training, Education, and the Quality Assessment of CD4+ T Lymphocyte Counting (I). Cytometry B Clin Cytom (2012) 82(2):93–100. doi: 10.1002/cyto.b.20624 21998025 PMC4126193

[207] Edwards BS Altobelli KK Nolla HA Harper DA Hoffman RR. Comprehensive Quality Assessment Approach for Flow Cytometric Immunophenotyping of Human Lymphocytes. Cytometry (1989) 10(4):433–41. doi: 10.1002/cyto.990100411 2788566

[208] Dimitrova E Taskov H Pashov A. Reproducibility of Estimation of CD3, CD4 and CD8 Reference Ranges Using Different Monoclonal Antibodies. Biologicals (1993) 21(3):215–20. doi: 10.1006/biol.1993.1078 8117435

[209] Pandolfi F Alario C Girardi E Rava L Ippolito G Kunkl A. The Italian Quality Control Study for Evaluation of CD4 Cells in Centres Involved in the Treatment of HIV-1 Patients. Italian CD4 Quality Control Group. Clin Exp Immunol (1998) 111(3):564–73. doi: 10.1046/j.1365-2249.1998.00520.x PMC1904887 9528900

[210] Whitby L Granger V Storie I Goodfellow K Sawle A Reilly JT. Quality Control of CD4+ T-Lymphocyte Enumeration: Results From the Last 9 Years of the United Kingdom National External Quality Assessment Scheme for Immune Monitoring (1993-2001). Cytometry (2002) 50(2):102–10. doi: 10.1002/cyto.10094 12116352

[211] Glencross DK Aggett HM Stevens WS Mandy F. African Regional External Quality Assessment for CD4 T-Cell Enumeration: Development, Outcomes, and Performance of Laboratories. Cytometry B Clin Cytom (2008) 74:S69–79. doi: 10.1002/cyto.b.20397 18228560

[212] Glencross DK Coetzee LM. Categorizing and Establishing CD4 Service Equivalency: Testing of Residual, Archived External Quality Assessment Scheme Sample Panels Enables Accelerated Virtual Peer Laboratory Review. Cytometry B Clin Cytom (2019) 96(5):404–16. doi: 10.1002/cyto.b.21772 30821061

[213] Yibalih NK Wolday D Kinde S Weldearegay GM. External Quality Assessment on CD4+ T-Cell Count Using in-House Proficiency Testing Panels for CD4 Count Laboratories in Addis Ababa, Ethiopia. Ethiop J Health Sci (2019) 29(3):309–20. doi: 10.4314/ejhs.v29i3.3 PMC6689725 31447499

[214] Pobkeeree V Lerdwana S Siangphoe U Noulsri E Polsrila K Nookhai S. External Quality Assessment Program on CD4+ T-Lymphocyte Counts for Persons With HIV/AIDS in Thailand: History and Accomplishments. Asian Pac J Allergy Immunol (2009) 27(4):225–32. 20232577

[215] Meyers AFA Bergeron M Thakar M Ding T Martel A Sandstrom P. QASI: A Collaboration for Implementation of an Independent Quality Assessment Programme in India. Afr J Lab Med (2016) 5(2):442. doi: 10.4102/ajlm.v5i2.442 28879123 PMC5433822

[216] Gaspar PC Wohlke BLP Brunialti MKC Pires AF Kohiyama IM Salomão R. External Quality Assessment for CD4 + T-Lymphocyte Count Test: Performance of the Brazilian Public Health Laboratories Network. Med (Baltimore) (2018) 97(1S Suppl 1):S32–7. doi: 10.1097/MD.0000000000010125 PMC5991543 29794603

[217] Koch U Radtke F. Mechanisms of T Cell Development and Transformation. Annu Rev Cell Dev Biol (2011) 27:539–62. doi: 10.1146/annurev-cellbio-092910-154008 21740230

[218] Mahnke YD Brodie TM Sallusto F Roederer M Lugli E. The Who’s Who of T-Cell Differentiation: Human Memory T-Cell Subsets. Eur J Immunol (2013) 43(11):2797–809. doi: 10.1002/eji.201343751 24258910

[219] Sallusto F Lenig D Förster R Lipp M Lanzavecchia A. Two Subsets of Memory T Lymphocytes With Distinct Homing Potentials and Effector Functions. Nature (1999) 401(6754):708–12. doi: 10.1038/44385 10537110

[220] Appay V van Lier RA Sallusto F Roederer M. Phenotype and Function of Human T Lymphocyte Subsets: Consensus and Issues. Cytometry A (2008) 73(11):975–83. doi: 10.1002/cyto.a.20643 18785267

[221] Monteseirín J Llamas E Bobadilla P Delgado J Jiménez MJ Conde J. The Study Cellular Subpopulations in Peripheral Blood From a Normal Reference Group Population (Blood Donors). Allergol Immunopathol (Madr) (1992) 20(1):9–12. 1380768

[222] McCloskey TW Cavaliere T Bakshi S Harper R Fagin J Kohn N. Immunophenotyping of T Lymphocytes by Three-Color Flow Cytometry in Healthy Newborns, Children, and Adults. Clin Immunol Immunopathol (1997) 84(1):46–55. doi: 10.1006/clin.1997.4370 9191883

[223] Huenecke S Behl M Fadler C Zimmermann SY Bochennek K Tramsen L. Age-Matched Lymphocyte Subpopulation Reference Values in Childhood and Adolescence: Application of Exponential Regression Analysis. Eur J Haematol (2008) 80(6):532–9. doi: 10.1111/j.1600-0609.2008.01052.x 18284628

[224] Provinciali M Moresi R Donnini A Lisa RM. Reference Values for CD4+ and CD8+ T Lymphocytes With Naïve or Memory Phenotype and Their Association With Mortality in the Elderly. Gerontology (2009) 55(3):314–21. doi: 10.1159/000199451 19190395

[225] van Gent R van Tilburg CM Nibbelke EE Otto SA Gaiser JF Janssens-Korpela PL. Refined Characterization and Reference Values of the Pediatric T- and B-Cell Compartments. Clin Immunol (2009) 133(1):95–107. doi: 10.1016/j.clim.2009.05.020 19586803

[226] Schatorjé EJ Gemen EF Driessen GJ Leuvenink J van Hout RW de Vries E. Paediatric Reference Values for the Peripheral T Cell Compartment. Scand J Immunol (2012) 75(4):436–44. doi: 10.1111/j.1365-3083.2012.02671.x 22420532

[227] Torres AJ Cisneiros P Guedes R Grassi MF Meyer R Bendicho MT. Lymphocyte Subset Reference Intervals in Blood Donors From Northeastern Brazil. Acad Bras Cienc (2015) 87(2):1019–25. doi: 10.1590/0001-3765201520130114 25923166

[228] Sahmoudi K El Allam A El Fakihi S Tahoune H Sadak A El Hafidi N. Moroccan Lymphocyte Subsets Reference Ranges: Age, Gender, Ethnicity, and Socio-Economic Factors Dependent Differences. J Immunoassay Immunochem (2020) 41(3):281–96. doi: 10.1080/15321819.2020.1728543 32065027

[229] Moraes-Pinto MI Ono E Santos-Valente EC Almeida LC Andrade PR Dinelli MI. Lymphocyte Subsets in Human Immunodeficiency Virus-Unexposed Brazilian Individuals From Birth to Adulthood. Mem Inst Oswaldo Cruz (2014) 109(8):989–98. doi: 10.1590/0074-0276140182 PMC4325616 25424448

[230] Bretschneider I Clemente MJ Meisel C Guerreiro M Streitz M Hopfenmüller W. Discrimination of T-Cell Subsets and T-Cell Receptor Repertoire Distribution. Immunol Res (2014) 58(1):20–7. doi: 10.1007/s12026-013-8473-0 24272857

[231] Botafogo V Pérez-Andres M Jara-Acevedo M Bárcena P Grigore G Hernández-Delgado A. Age Distribution of Multiple Functionally Relevant Subsets of CD4+ T Cells in Human Blood Using a Standardized and Validated 14-Color EuroFlow Immune Monitoring Tube. Front Immunol (2020) 11:166. doi: 10.3389/fimmu.2020.00166 32174910 PMC7056740

[232] Flanagan SE Haapaniemi E Russell MA Caswell R Allen HL De Franco E. Activating Germline Mutations in STAT3 Cause Early-Onset Multi-Organ Autoimmune Disease. Nat Genet (2014) 46(8):812–4. doi: 10.1038/ng.3040 PMC4129488 25038750

[233] Delmonte OM Fleisher TA. Flow Cytometry: Surface Markers and Beyond. J Allergy Clin Immunol (2019) 143(2):528–37. doi: 10.1016/j.jaci.2018.08.011 30170120

[234] Chiang SCC Vergamini SM Husami A Neumeier L Quinn K Ellerhorst T. Screening for Wiskott-Aldrich Syndrome by Flow Cytometry. J Allergy Clin Immunol (2018) 142(1):333–5.e8. doi: 10.1016/j.jaci.2018.04.017 29729304

[235] Rawat A Arora K Shandilya J Vignesh P Suri D Kaur G. Flow Cytometry for Diagnosis of Primary Immune Deficiencies-A Tertiary Center Experience From North India. Front Immunol (2019) 10:2111. doi: 10.3389/fimmu.2019.02111 31572360 PMC6749021

[236] Rozmus J Junker A Thibodeau ML Grenier D Turvey SE Yacoub W. Severe Combined Immunodeficiency (SCID) in Canadian Children: A National Surveillance Study. J Clin Immunol (2013) 33(8):1310–6. doi: 10.1007/s10875-013-9952-8 PMC7102302 24122030

[237] Shahbazi Z Yazdani R Shahkarami S Shahbazi S Hamid M Sadeghi-Shabestari M. Genetic Mutations and Immunological Features of Severe Combined Immunodeficiency Patients in Iran. Immunol Lett (2019) 216:70–8. doi: 10.1016/j.imlet.2019.10.001 31589898

[238] Purswani P Meehan CA Kuehn HS Chang Y Dasso JF Meyer AK. Two Unique Cases of X-Linked SCID: A Diagnostic Challenge in the Era of Newborn Screening. Front Pediatr (2019) 7:55. doi: 10.3389/fped.2019.00055 31024866 PMC6460992

[239] Oliveira JB Notarangelo LD Fleisher TA. Applications of Flow Cytometry for the Study of Primary Immune Deficiencies. Curr Opin Allergy Clin Immunol (2008) 8(6):499–509. doi: 10.1097/ACI.0b013e328312c790 18978463

[240] Furukawa H Yabe T Watanabe K Miyamoto R Miki A Akaza T. Tolerance of NK and LAK Activity for HLA Class I-Deficient Targets in a TAP1-Deficient Patient (Bare Lymphocyte Syndrome Type I). Hum Immunol (1999) 60(1):32–40. doi: 10.1016/S0198-8859(98)00097-4 9952025

[241] Kallen ME Pullarkat ST. Type II Bare Lymphocyte Syndrome: Role of Peripheral Blood Flow Cytometry and Utility of Stem Cell Transplant in Treatment. J Pediatr Hematol Oncol (2015) 37(4):e245–9. doi: 10.1097/MPH.0000000000000278 25354255

[242] Jacobsen M Hoffmann S Cepok S Stei S Ziegler A Sommer N. A Novel Mutation in PTPRC Interferes With Splicing and Alters the Structure of the Human CD45 Molecule. Immunogenetics (2002) 54(3):158–63. doi: 10.1007/s00251-002-0455-7 12073144

[243] Pai SY de Boer H Massaad MJ Chatila TA Keles S Jabara HH. Flow Cytometry Diagnosis of Dedicator of Cytokinesis 8 (DOCK8) Deficiency. J Allergy Clin Immunol (2014) 134(1):221–3. doi: 10.1016/j.jaci.2014.02.023 PMC6317337 24698323

[244] Bogaert DJ Kuehn HS Bonroy C Calvo KR Dehoorne J Vanlander AV. A Novel IKAROS Haploinsufficiency Kindred With Unexpectedly Late and Variable B-Cell Maturation Defects. J Allergy Clin Immunol (2018) 141(1):432–5.e7. doi: 10.1016/j.jaci.2017.08.019 28927821 PMC6588539

[245] Arredondo-Vega FX Santisteban I Daniels S Toutain S Hershfield MS. Adenosine Deaminase Deficiency: Genotype-Phenotype Correlations Based on Expressed Activity of 29 Mutant Alleles. Am J Hum Genet (1998) 63(4):1049–59. doi: 10.1086/302054 PMC1377486 9758612

[246] Cagdas D Gur Cetinkaya P Karaatmaca B Esenboga S Tan C Yılmaz T. ADA Deficiency: Evaluation of the Clinical and Laboratory Features and the Outcome. J Clin Immunol (2018) 38(4):484–93. doi: 10.1007/s10875-018-0496-9 29744787

[247] Scott CR Chen SH Giblett ER. Detection of the Carrier State in Combined Immunodeficiency Disease Associated With Adenosine Deaminase Deficiency. J Clin Invest (1974) 53(4):1194–6. doi: 10.1172/JCI107658 PMC333106 4815083

[248] Pollara B Meuwissen HJ. Letter: Combined Immunodeficiency Disease and A.D.A. Deficiency. Lancet (1973) 2(7841):1324. doi: 10.1016/s0140-6736(73)92897-3 4127665

[249] Meuwissen HJ Pickering RJ Pollara B. Adenosine Deaminase Deficiency in Combined Immunologic Deficiency Disease. Birth Defects Orig Artic Ser (1975) 11(1):117–9. 1148375

[250] Pollara B Moore JJ Pickering RJ Gabrielsen AE Meuwissen HJ. Combined Immunodeficiency Disease: An Inborn Error of Purine Metabolism. Birth Defects Orig Artic Ser (1975) 11(1):120–3. 1148376

[251] Hirschhorn R Roegner V Jenkins T Seaman C Piomelli S Borkowsky W. Erythrocyte Adenosine Deaminase Deficiency Without Immunodeficiency. Evidence for an Unstable Mutant Enzyme. J Clin Invest (1979) 64(4):1130–9. doi: 10.1172/JCI109552 PMC372225 479373

[252] Jenkins T Lane AB Nurse GT Hopkinson DA. Red Cell Adenosine Deaminase (ADA) Polymorphism in Southern Africa, With Special Reference to ADA Deficiency Among the!Kung. Ann Hum Genet (1979) 42(4):425–33. doi: 10.1111/j.1469-1809.1979.tb00676.x 475331

[253] Storch H Krüger W Rotzsch W. Adenosine Deaminase Activity in Plasma and Blood Cells of Patients With Haematological and Autoimmune Diseases. Acta Haematol (1981) 65(3):183–8. doi: 10.1159/000207176 6785972

[254] Agarwal RP Crabtree GW Parks RE Nelson JA Keightley R Parkman R. Purine Nucleoside Metabolism in the Erythrocytes of Patients With Adenosine Deaminase Deficiency and Severe Combined Immunodeficiency. J Clin Invest (1976) 57(4):1025–35. doi: 10.1172/JCI108344 PMC436746 947948

[255] la Marca G Giocaliere E Malvagia S Villanelli F Funghini S Ombrone D. Development and Validation of a 2nd Tier Test for Identification of Purine Nucleoside Phosphorylase Deficiency Patients During Expanded Newborn Screening by Liquid Chromatography-Tandem Mass Spectrometry. Clin Chem Lab Med (2016) 54(4):627–32. doi: 10.1515/cclm-2015-0436 26466166

[256] Fossati P. Phosphate Determination by Enzymatic Colorimetric Assay. Anal Biochem (1985) 149(1):62–5. doi: 10.1016/0003-2697(85)90476-2 3935004

[257] Chan AY Leiding JW Liu X Logan BR Burroughs LM Allenspach EJ. Hematopoietic Cell Transplantation in Patients With Primary Immune Regulatory Disorders (PIRD): A Primary Immune Deficiency Treatment Consortium (PIDTC) Survey. Front Immunol (2020) 11:239. doi: 10.3389/fimmu.2020.00239 32153572 PMC7046837

[258] Morimoto A Nakazawa Y Ishii E. Hemophagocytic Lymphohistiocytosis: Pathogenesis, Diagnosis, and Management. Pediatr Int (2016) 58(9):817–25. doi: 10.1111/ped.13064 27289085

[259] Damoiseaux J. The IL-2 - IL-2 Receptor Pathway in Health and Disease: The Role of the Soluble IL-2 Receptor. Clin Immunol (2020) 218:108515. doi: 10.1016/j.clim.2020.108515 32619646

[260] Lin M Park S Hayden A Giustini D Trinkaus M Pudek M. Clinical Utility of Soluble Interleukin-2 Receptor in Hemophagocytic Syndromes: A Systematic Scoping Review. Ann Hematol (2017) 96(8):1241–51. doi: 10.1007/s00277-017-2993-y 28497365

[261] Bien E Balcerska A. Serum Soluble Interleukin 2 Receptor Alpha in Human Cancer of Adults and Children: A Review. Biomarkers (2008) 13(1):1–26. doi: 10.1080/13547500701674063 17906988

[262] Hayden A Lin M Park S Pudek M Schneider M Jordan MB. Soluble Interleukin-2 Receptor is a Sensitive Diagnostic Test in Adult HLH. Blood Adv (2017) 1(26):2529–34. doi: 10.1182/bloodadvances.2017012310 PMC5728644 29296904

[263] Gotoh Y Okamoto Y Uemura O Mori N Tanaka S Ando T. Determination of Age-Related Changes in Human Soluble Interleukin 2 Receptor in Body Fluids of Normal Subjects as a Control Value Against Disease States. Clin Chim Acta (1999) 289(1-2):89–97. doi: 10.1016/S0009-8981(99)00161-8 10556656

[264] Bharwani KD Dirckx M Stronks DL Dik WA Schreurs MWJ Huygen FJPM. Elevated Plasma Levels of sIL-2R in Complex Regional Pain Syndrome: A Pathogenic Role for T-Lymphocytes? Mediators Inflammation (2017) 2017:2764261. doi: 10.1155/2017/2764261 PMC5467333 28634419

[265] Henter JI Horne A Aricó M Egeler RM Filipovich AH Imashuku S. HLH-2004: Diagnostic and Therapeutic Guidelines for Hemophagocytic Lymphohistiocytosis. Pediatr Blood Cancer (2007) 48(2):124–31. doi: 10.1002/pbc.21039 16937360

[266] Ham H Billadeau DD. Human Immunodeficiency Syndromes Affecting Human Natural Killer Cell Cytolytic Activity. Front Immunol (2014) 5:2. doi: 10.3389/fimmu.2014.00002 24478771 PMC3896857

[267] Chiang SCC Bleesing JJ Marsh RA. Current Flow Cytometric Assays for the Screening and Diagnosis of Primary HLH. Front Immunol (2019) 10:1740. doi: 10.3389/fimmu.2019.01740 31396234 PMC6664088

[268] Risma KA Marsh RA. Hemophagocytic Lymphohistiocytosis: Clinical Presentations and Diagnosis. J Allergy Clin Immunol Pract (2019) 7(3):824–32. doi: 10.1016/j.jaip.2018.11.050 30557712

[269] Rubin TS Zhang K Gifford C Lane A Choo S Bleesing JJ. Perforin and CD107a Testing is Superior to NK Cell Function Testing for Screening Patients for Genetic HLH. Blood (2017) 129(22):2993–9. doi: 10.1182/blood-2016-12-753830 PMC5766842 28270454

[270] Gifford CE Weingartner E Villanueva J Johnson J Zhang K Filipovich AH. Clinical Flow Cytometric Screening of SAP and XIAP Expression Accurately Identifies Patients With SH2D1A and XIAP/BIRC4 Mutations. Cytometry B Clin Cytom (2014) 86(4):263–71. doi: 10.1002/cytob.21166 24616127

[271] Ammann S Elling R Gyrd-Hansen M Dückers G Bredius R Burns SO. A New Functional Assay for the Diagnosis of X-Linked Inhibitor of Apoptosis (XIAP) Deficiency. Clin Exp Immunol (2014) 176(3):394–400. doi: 10.1111/cei.12306 24611904 PMC4008984

[272] Fleisher TA Oliveira JB. Monogenic Defects in Lymphocyte Apoptosis. Curr Opin Allergy Clin Immunol (2012) 12(6):609–15. doi: 10.1097/ACI.0b013e3283588da0 22918222

[273] Price S Shaw PA Seitz A Joshi G Davis J Niemela JE. Natural History of Autoimmune Lymphoproliferative Syndrome Associated With FAS Gene Mutations. Blood (2014) 123(13):1989–99. doi: 10.1182/blood-2013-10-535393 PMC3968385 24398331

[274] Oliveira JB Bleesing JJ Dianzani U Fleisher TA Jaffe ES Lenardo MJ. Revised Diagnostic Criteria and Classification for the Autoimmune Lymphoproliferative Syndrome (ALPS): Report From the 2009 NIH International Workshop. Blood (2010) 116(14):e35–40. doi: 10.1182/blood-2010-04-280347 PMC2953894 20538792

[275] Bleesing JJ Brown MR Straus SE Dale JK Siegel RM Johnson M. Immunophenotypic Profiles in Families With Autoimmune Lymphoproliferative Syndrome. Blood (2001) 98(8):2466–73. doi: 10.1182/blood.V98.8.2466 11588044

[276] Bleesing JJ Brown MR Dale JK Straus SE Lenardo MJ Puck JM. TcR-Alpha/Beta(+) CD4(-)CD8(-) T Cells in Humans With the Autoimmune Lymphoproliferative Syndrome Express a Novel CD45 Isoform That Is Analogous to Murine B220 and Represents a Marker of Altered O-Glycan Biosynthesis. Clin Immunol (2001) 100(3):314–24. doi: 10.1006/clim.2001.5069 11513545

[277] Magerus-Chatinet A Stolzenberg MC Loffredo MS Neven B Schaffner C Ducrot N. FAS-L, IL-10, and Double-Negative CD4- CD8- TCR Alpha/Beta+ T Cells are Reliable Markers of Autoimmune Lymphoproliferative Syndrome (ALPS) Associated With FAS Loss of Function. Blood (2009) 113(13):3027–30. doi: 10.1182/blood-2008-09-179630 19176318

[278] Caminha I Fleisher TA Hornung RL Dale JK Niemela JE Price S. Using Biomarkers to Predict the Presence of FAS Mutations in Patients With Features of the Autoimmune Lymphoproliferative Syndrome. J Allergy Clin Immunol (2010) 125(4):946–9.e6. doi: 10.1016/j.jaci.2009.12.983 20227752 PMC3412519

[279] Santegoets SJ Dijkgraaf EM Battaglia A Beckhove P Britten CM Gallimore A. Monitoring Regulatory T Cells in Clinical Samples: Consensus on an Essential Marker Set and Gating Strategy for Regulatory T Cell Analysis by Flow Cytometry. Cancer Immunol Immunother (2015) 64(10):1271–86. doi: 10.1007/s00262-015-1729-x PMC4554737 26122357

[280] Akimova T Levine MH Beier UH Hancock WW. Standardization, Evaluation, and Area-Under-Curve Analysis of Human and Murine Treg Suppressive Function. Methods Mol Biol (2016) 1371:43–78. doi: 10.1007/978-1-4939-3139-2_4 26530794 PMC5554116

[281] Pitoiset F Barbié M Monneret G Braudeau C Pochard P Pellegrin I. A Standardized Flow Cytometry Procedure for the Monitoring of Regulatory T Cells in Clinical Trials. Cytometry B Clin Cytom (2018) 94(5):621–6. doi: 10.1002/cyto.b.21622 29316248

[282] Manuszak C Brainard M Thrash E Hodi FS Severgnini M. Standardized 11-Color Flow Cytometry Panel for the Functional Phenotyping of Human T Regulatory Cells. J Biol Methods (2020) 7(2):e131. doi: 10.14440/jbm.2020.325 32313815 PMC7163208

[283] Kim H Moon HW Hur M Park CM Yun YM Hwang HS. Distribution of CD4+ CD25 High FoxP3+ Regulatory T-Cells in Umbilical Cord Blood. J Matern Fetal Neonatal Med (2012) 25(10):2058–61. doi: 10.3109/14767058.2012.666591 22348656

[284] Rennó C Nadaf MI Zago CA Carneiro-Sampaio M Palmeira P. Healthy Preterm Newborns Show an Increased Frequency of CD4(+) CD25(high) CD127(low) FOXP3(+) Regulatory T Cells With a Naive Phenotype and High Expression of Gut-Homing Receptors. Scand J Immunol (2016) 83(6):445–55. doi: 10.1111/sji.12435 27007547

[285] Bustamante J. Mendelian Susceptibility to Mycobacterial Disease: Recent Discoveries. Hum Genet (2020) 139(6-7):993–1000. doi: 10.1007/s00439-020-02120-y 32025907 PMC7275902

[286] Sologuren I Boisson-Dupuis S Pestano J Vincent QB Fernández-Pérez L Chapgier A. Partial Recessive IFN-γr1 Deficiency: Genetic, Immunological and Clinical Features of 14 Patients From 11 Kindreds. Hum Mol Genet (2011) 20(8):1509–23. doi: 10.1093/hmg/ddr029 PMC3115578 21266457

[287] Jouanguy E Lamhamedi-Cherradi S Lammas D Dorman SE Fondanèche MC Dupuis S. A Human IFNGR1 Small Deletion Hotspot Associated With Dominant Susceptibility to Mycobacterial Infection. Nat Genet (1999) 21(4):370–8. doi: 10.1038/7701 10192386

[288] Fleisher TA Dorman SE Anderson JA Vail M Brown MR Holland SM. Detection of Intracellular Phosphorylated STAT-1 by Flow Cytometry. Clin Immunol (1999) 90(3):425–30. doi: 10.1006/clim.1998.4654 10075873

[289] de Beaucoudrey L Samarina A Bustamante J Cobat A Boisson-Dupuis S Feinberg J. Revisiting Human IL-12rβ1 Deficiency: A Survey of 141 Patients From 30 Countries. Med (Baltimore) (2010) 89(6):381–402. doi: 10.1097/MD.0b013e3181fdd832 PMC3129625 21057261

[290] Uzel G Frucht DM Fleisher TA Holland SM. Detection of Intracellular Phosphorylated STAT-4 by Flow Cytometry. Clin Immunol (2001) 100(3):270–6. doi: 10.1006/clim.2001.5078 11513540

[291] Sullivan KE. Defects in Adhesion Molecules. Clin Rev Allergy Immunol (2000) 19(2):109–25. doi: 10.1385/CRIAI:19:2:109 11107497

[292] Etzioni A. Genetic Etiologies of Leukocyte Adhesion Defects. Curr Opin Immunol (2009) 21(5):481–6. doi: 10.1016/j.coi.2009.07.005 19647987

[293] A E JM H. Cell Adhesion and Leukocyte Adhesion Defects. In: Ochs H, editor. Primary Immunodeficiency Diseases: A Molecular and Genetic Approach, 1 ed. New York: Oxford University Press (1999). p. 375–88.

[294] Anderson DC Springer TA. Leukocyte Adhesion Deficiency: An Inherited Defect in the Mac-1, LFA-1, and P150,95 Glycoproteins. Annu Rev Med (1987) 38:175–94. doi: 10.1146/annurev.me.38.020187.001135 3555290

[295] Verma NK Kelleher D. Not Just an Adhesion Molecule: LFA-1 Contact Tunes the T Lymphocyte Program. J Immunol (2017) 199(4):1213–21. doi: 10.4049/jimmunol.1700495 28784685

[296] Bissenden JG Haeney MR Tarlow MJ Thompson RA. Delayed Separation of the Umbilical Cord, Severe Widespread Infections, and Immunodeficiency. Arch Dis Child (1981) 56(5):397–9. doi: 10.1136/adc.56.5.397 PMC1627445 7259263

[297] Lühn K Wild MK Eckhardt M Gerardy-Schahn R Vestweber D. The Gene Defective in Leukocyte Adhesion Deficiency II Encodes a Putative GDP-Fucose Transporter. Nat Genet (2001) 28(1):69–72. doi: 10.1038/ng0501-69 11326279

[298] Svensson L Howarth K McDowall A Patzak I Evans R Ussar S. Leukocyte Adhesion Deficiency-III is Caused by Mutations in KINDLIN3 Affecting Integrin Activation. Nat Med (2009) 15(3):306–12. doi: 10.1038/nm.1931 PMC2680140 19234463

[299] Harris ES Weyrich AS Zimmerman GA. Lessons From Rare Maladies: Leukocyte Adhesion Deficiency Syndromes. Curr Opin Hematol (2013) 20(1):16–25. doi: 10.1097/MOH.0b013e32835a0091 23207660 PMC3564641

